# Translation, cultural adaptation, and psychometric evaluation of the Patient Assessment Chronic Illness Care tool in Ethiopia (PACIC-5As-ET) for patients with type 2 diabetes

**DOI:** 10.1371/journal.pone.0329197

**Published:** 2026-06-11

**Authors:** Yohannes Mulu Ferede, Margareta Westerbotn, Mignote Hailu Gebrie, Debrework Tesgera Beshah, Kerstin Erlandsson

**Affiliations:** 1 Department of Medical Nursing, School of Nursing, College of Medicine and Health Sciences, University of Gondar, Gondar, Ethiopia; 2 Institution of Nursing Science, Sophiahemmet University, Stockholm, Sweden; 3 School of Nursing, College of Medicine and Health Sciences, University of Gondar, Gondar, Ethiopia; 4 School of Health and Welfare, Dalarna University, Falun, Sweden; STIKES Wira Medika PPNI Bali: Sekolah Tinggi Ilmu Kesehatan Wira Medika PPNI Bali, INDONESIA

## Abstract

**Introduction:**

Chronic conditions are a significant global health challenge that adversely affects the quality of care for patients with type 2 diabetes (T2D). To evaluate and improve the quality of care, the Patient Assessment Chronic Illness Care (PACIC-5As) tool has been developed. It is the most widely used tool designed to assess the perceived quality of care among individuals with chronic conditions, including diabetes. Nevertheless, it has not yet been culturally adapted and validated in the Ethiopian context. Therefore, this study aimed to translate, culturally adapt, and evaluate the psychometric properties of the PACIC-5As tool in Ethiopia.

**Methods:**

A multicenter cross-sectional study was conducted among individuals with type 2 diabetes from March 24, 2025, to May 5, 2025, in the Amhara region’s comprehensive specialized referral hospitals. A systematic random sampling technique was used to select the study participants. Data were collected through face-to-face interviews. The tool consists of 26 items and 5 domains. Content validity was assessed at both the individual and scale levels. Internal consistency was evaluated using Cronbach’s alpha (α) and composite reliability (CR), with a value ≥ 0.70 considered acceptable. Confirmatory factor analysis (CFA) was conducted to evaluate model fit and factor structure. Model fit was assessed using the absolute and incremental fit indices and interpreted based on the recommended thresholds. Convergent validity was computed using average variance extracted (AVE), with a value ≥ 0.4 considered adequate, while discriminant validity was evaluated using AVE and inter-construct correlations.

**Results:**

A total of 520 study participants were enrolled, and 517 (99.4%) were included in the study. The overall mean summary score of PACIC-5As-ET was 2.68 (±0.62). The content validity index at the item and scale levels ranged from 80% to 100%, with an inter-rater agreement of 95%. The Cronbach’s alpha and composite reliability (CR) of the PACIC-5As-ET were 0.93. The Cronbach’s alpha values for the subscales ranged from 0.71 (Assist) to 0.82 (Arrange). The test-retest reliability of PACIC-5As-ET was 0.94. The model fit indices were χ²/df (2.79), RMSEA (0.06), SRMR (0.08), GFI (0.89), and CFI (0.40). The AVE value of the overall PACIC-5As-ET was 0.93, and the subscales ranged from 0.47 (Advise) to 0.59 (Arrange).

**Conclusions:**

The Amharic version of the PACIC-5As-ET tool demonstrated excellent internal consistency and acceptable validity for assessing the perceptions of patients with T2D. The absolute fit indices were generally within the recommended range, whereas the incremental fit indices were low. Therefore, support for the hypothesized five-factor structure is limited and should be interpreted with caution.

## Introduction

Type 2 diabetes (T2D) is a global pandemic disease characterized by hyperglycemia resulting from reduced insulin secretion, resistance, or both [1]. Globally, around 462 million people are living with T2D, accounting for more than 95% of all diabetes cases [2–6]. Nearly 80% of these cases are in low- and middle-income countries [7]; in Africa, the proportion ranges from 70% to 90% [8]; in sub-Saharan Africa, the reported prevalence is 5% [9]; and in Ethiopia, it ranges from 5.8% to 7.3% [10].

Patients with T2D gradually experience chronic hyperglycemia, which can lead to the development of comorbid conditions [11]. The likelihood of developing chronic comorbid conditions is at least twofold higher among individuals with T2D compared with those without diabetes [12–14]. This increases the prevalence of chronic conditions among individuals with T2D [15]. As a result, the presence of these multiple health problems among individuals with T2D increases the demand for chronic care management within the healthcare system [16]. Thus, the quality of care is often inadequate and fails to meet the complex needs of individuals with T2D, particularly patients with comorbid conditions [17]. Furthermore, many healthcare systems prioritize the management of individual diseases instead of delivering comprehensive, patient-centered care that takes into account the complexities associated with various chronic conditions [18]. Consequently, care becomes fragmented, leading to confusion, increased financial and treatment burdens, and poor health outcomes [19–21].

To enhance the quality of care as well as promote patient-centered care, it is essential to actively involve patients in their care. Hence, the increasing role of patients in shaping healthcare quality has led to a greater emphasis on incorporating their feedback into service delivery [22]. Therefore, patient involvement is crucial for achieving patient-centered care [23].

Patient-centered care focuses on meeting each person's unique needs, preferences, and values, guiding clinical decisions [23,24]. Moreover, encouraging patient participation in healthcare delivery improves treatment outcomes, strengthens communication within healthcare teams, optimizes satisfaction, increases the productivity of service providers, and facilitates the evaluation of services from the patients’ perspectives [23–27]. Patient experience or perception is a key outcome of patient-centered care, which involves patients and healthcare providers collaborating to make informed decisions, address individual needs and preferences, and ensure that care is respectful, responsive, and tailored to each patient [23,28].

As a result, to effectively evaluate the care provided to patients with T2D, the measurement tool must be culturally adapted and scientifically valid for the population being assessed or studied [29]. Hence, the PACIC-5As tool was developed to assess the perceived quality of care among individuals living with chronic disease, including diabetes. This tool evaluates the extent to which patients receive health care services that align with the Chronic Care Model (CCM) [30], and it is considered the most comprehensive and relevant tool for evaluating patient-centered and integrated care from the patient’s perspective [31]. The tool was originally developed by Glasgow et al. (2005) in the USA [32] and further validated in Australia [33], Saudi Arabia [34], Iran [35], the Netherlands [36], Thailand [37], Greek [38], and India [39] among patients with T2D.

However, in Ethiopia, the PACIC-5A tool has not yet been adapted and validated. Moreover, there are limited studies evaluating patient-centered care in Ethiopian public hospitals [40], which may indicate a substantial gap in the delivery of quality care for patients with T2D. Therefore, there is a need to evaluate and improve the care delivery for patients with T2D using a well-designed and scientifically valid tool. This study supports researchers, policymakers, and healthcare providers in identifying gaps and implementing appropriate interventions for patients with T2D. Therefore, this study aimed to culturally translate, adapt, and evaluate the psychometric properties of the PACIC-5As instrument in Ethiopia for patients with T2D.

## Methods and materials

### Study design, period, and setting

A multicenter, cross-sectional study was conducted in the Amhara region's comprehensive specialized referral hospitals (CSRHs) from March 24, 2025, to May 5, 2025. The Amhara region is the second-largest region in Ethiopia, located in the northwestern part of the country. According to the 2007 census, the estimated total population of the Amhara region was 17,221,976 (male = 8,641,580 & female = 8,580,396) [41].

The region is served by eight comprehensive specialized referral hospitals, two general hospitals, 73 primary hospitals, 847 health centers, and 3,342 health posts. The eight public comprehensive specialized referral hospitals are the University of Gondar Comprehensive Specialized Referral Hospital (UGCSRH), Bahir Dar Felege Hiwot Comprehensive Specialized Referral Hospital (BFCSRH), Bahir Dar Tibebe Gion Comprehensive Specialized Referral Hospital (BTCSRH), Debere Markos Comprehensive Specialized Referral Hospital (DMCSRH), Debere Tabor Comprehensive Specialized Referral Hospital (DTCSRH), Debere Bihrhan Comprehensive Specialized Referral Hospital (DBCSRH), Dessie Comprehensive Specialized Referral Hospital (DCSRH), and Woldia Comprehensive Specialized Referral Hospital (WCSRH). Based on the information from the regional health bureau and the hospital health management information system (HMIS) report, the annual average number of outpatient follow-ups for T2D was 1,297 [42].

### Source and study population

All adults with T2D who had outpatient follow-up care at CSRHs in the Amhara region, Ethiopia, constituted the source population, whereas those who attended during the data collection period in the randomly selected CSRHs were the study population.

### Inclusion and exclusion criteria

All individuals aged 18 years or older diagnosed with T2D who had at least 6 months of outpatient follow-up care and who were attending during the data collection period were included in the study.

### Sample size determination

The sample size for confirmatory factor analysis (CFA) or structural equation modeling (SEM) was determined using the recommended parameter-to-sample ratio, which ranges from 1:5–1:20 [43]. Based on this guideline, we used the 1:20 parameter-to-sample ratio, resulting in a total sample size of 520.

### Sampling procedure

Out of eight comprehensive specialized referral hospitals, three hospitals were randomly selected. These were Debre Markos Comprehensive Specialized Referral Hospital (DMSCRH), Bahir Dar Felege Hiwot Comprehensive Specialized Referral Hospital (BFCSRH), and Woldia Comprehensive Specialized Referral Hospital (WCSRH). The sample size for each CSRH was determined using the proportional allocation formula $\left(ni=\frac{\text{n x Ni}}{N}\;\right)\;,$ where *ni* represents the sample size for a single hospital, *n* is the total sample size, *Ni* is the total population of a single hospital, and *N* is the total population across all eight hospitals. Participants were selected using a systematic random sampling technique. The sampling interval was calculated using the formula (K = N/n), where *N* represents the total population and *n* is the total sample size. Therefore, participants were selected at every K = 2 interval based on their order of arrival at the hospital during their follow-up period.

### Data collection tool and procedure

The Patient Assessment Chronic Illness Care (PACIC-5As) measure is the most widely used tool designed to assess the implementation of chronic illness care from the patient’s perspective [31,44]. The tool aligns with the concept of the Chronic Care Model (CCM) [30]. The PACIC tool was originally developed by Glasgow et al. (2005) [45] and subsequently expanded into the PACIC-5As by incorporating items that included concepts from the behavioral change model (behavioral counseling intervention). The tool demonstrated an internal consistency of 0.97 [32].

The 5As model is an evidence-based approach to behavioral change employed to improve patients’ self-management [46]. The PACIC-5As comprises 26 items, integrating the original PACIC instrument with the “5As” behavioral change model, which is recommended by the United States Preventive Services Task Force (USPSTF) [46]. The 5As model includes five subscales: Assess, Advise, Agree, Assist, and Arrange. “Assess” includes items 1, 11, 15, 20, and 21, which evaluates behavioral health risks, symptoms, or current behaviors. “Advise” contains items 4, 6, 9, 19, and 24, which provide clear, specific, and personalized behavioral counseling. “Agree” consists of items 2, 3, 7, 8, and 25, which focus on collaboratively selecting appropriate treatment goals and reaching a shared agreement. “Assist” includes items 10, 12, 13, 14, and 26, which helps or supports patients in making lifestyle changes. “Arrange” comprises items 16, 17, 18, 22, and 23, which are designed to assess ongoing assistance or follow-up care [46].

The items are scored on a 5-point Likert scale, where 1 represents “almost never” and 5 represents “almost always.” The overall PACIC-5As score (summary score) was obtained by averaging all items, excluding item 5 (1−4, 6−26). Each subscale score of the PACIC-5As was calculated by averaging the items corresponding to each subscale [45]. Higher PACIC-5As scores indicate better perceived quality of diabetes care [33,45,47].

Data were collected using Kobo Toolbox software version 4.4. Following the translation and validation process, the questionnaire was created and prepared in both English and Amharic languages within the software. The software is suitable for collecting data both online and offline. Subsequently, data were gathered through face-to-face interviews.

Prior to conducting the translation and validation process, permission was obtained from the tool developer, Glasgow et al. (2005) [45]. In this study, the translation, cultural adaptation, and validation process adhered to the guideline for the translation, adaptation, and validation of instruments for use in cross-cultural health care research [48]. This guideline consists of a series of stages, ranging from translation to psychometric evaluation. We followed this guideline and conducted the translation and adaptation process as follows:

**Stage I: Forward translation:** The forward translation was carried out by two bilingual translators whose native language is Amharic, the target language. Each translator independently translated the English PACIC-5As tool into Amharic. The first translator (FT-1) is an assistant professor of medical nursing at the University of Gondar, familiar with healthcare terminology and the instrument's content. The second translator (FT-2) is an assistant professor of teaching English as a foreign language (TEFL) at the University of Gondar. He possesses expertise in language and is familiar with the culture being studied, but he is not well-versed in healthcare terminology and the instrument's content. Both translators provided written reports highlighting challenging phrases and explaining their translation choices. This process included translating all components of the instrument, including item content, response options, and instructions.

**Stage II: Synthesis of the translation:** At this stage, the two translators (FT-1 and FT-2) compared and synthesized their translations through a committee approach. Any discrepancies or ambiguities in wording, sentences, and meanings between their translations were discussed. After discussion, both translators confirmed content equivalence and reached a consensus. The principal investigator also actively participated in the discussions, clarifying issues and supporting both translators in reaching an agreement. Finally, the preliminary translated Amharic version of the PACIC-5As-ET instrument (FT-12) was produced.

**Stage III: Back translation:** After completing the forward translation, two bilingual back translators, Back Translator 1 (BT-1) and Back Translator 2 (BT-2), independently translated the Amharic version of the PACIC-5As-ET tool back into English. BT-1 was an assistant professor in the Department of Medical Nursing at the University of Gondar and an expert in healthcare terminology and the conceptual content area of the instrument. BT-2 was an assistant professor of Teaching English as a Foreign Language (TEFL) at the University of Gondar. Whereas he understood the cultural and grammatical nuances of English, however, his familiarity with medical terminology and the instrument being studied was limited.

During the translation process, the back translators (BT-1 and BT-2) were unaware of the original instrument and did not have access to it (blind back translation). After completing the back translation, both translators convened to discuss and reconcile each question with the original English version. Any issues, including differences in translation, lack of clarity, or ambiguities in words and sentences, were thoroughly identified and resolved through mutual agreement. Additionally, the principal investigator actively participated in the discussions and solved the problem together.

**Stage IV: Expert committee:** At this stage, an expert panel discussion was conducted to ensure the cross-cultural equivalence of the newly translated PACIC-5As-ET instrument and to evaluate its content validity. A total of ten experts participated in the panel discussion, including one PhD holder in public health (a member of the research team), one PhD in nursing, and experts with MSc degrees in nutrition, midwifery, surgical nursing, and pediatric nursing. Both forward and back translators also participated in the panel discussion. All the experts had over ten years of experience in their respective fields.

The expert committee evaluated and reviewed the instructions, items, and response formats for any similarities or differences in meanings, wording, or grammatical structures between the forward translations (FT-1, FT-2, and FT-12) and the back translations (BT-1 and BT-2). All translations were then compared with the original questionnaire. The committee discussed any grammatical errors, ambiguities, discrepancies in meaning, or challenges in language construction and then reached a consensus. At this stage, initial conceptual, content, and semantic equivalence were established. Accordingly, the experts independently evaluated and rated the content of the pre-final version of the PACIC-5As-ET in terms of its relevance for measuring the perceived quality of care among patients with T2D. Finally, the committee approved the pre-final Amharic version of the PACIC-5As-ET tool for use in Ethiopia.

**Stage V: Pilot testing:** In this stage, the pre-final version of the newly translated and adapted PACIC-5As-ET measure was evaluated by participants. Approximately 20 patients with T2D were included in the pretest at the University of Gondar Comprehensive Specialized Referral Hospital.

**Stage VI:** After completing the content and face validity assessments, the psychometric properties of the newly translated and adapted PACIC-5As-ET tool were evaluated among 520 patients with T2D. This analysis included the assessment of the tool’s reliability, validity, model fit, and other psychometric properties.

### Data management and analysis

Following data collection, the data were reviewed, cleaned, and exported to Statistical Package for the Social Sciences (SPSS) version 25 for analysis. Additionally, Analysis of Moment Structures (AMOS) version 26 software was used to conduct confirmatory factor analysis (CFA). The results are presented using descriptive statistics, including mean (±SD), frequencies with percentages, figures, and tables.

### Content validity

After the panel discussion, all expert committee members independently evaluated and rated the pre-final version of the PACIC-5As-ET tool. The committee assessed the relevance of the tool using a four-point Likert scale: 1 (not relevant), 2 (somewhat relevant), 3 (quite relevant), and 4 (highly relevant). Items rated as 1 (not relevant) or 2 (somewhat relevant) were considered for revision. The scores were then dichotomized into relevant and not relevant categories. Ratings of 3 (quite relevant) and 4 (highly relevant) were categorized as relevant and scored as one, while ratings of 1 (not relevant) and 2 (somewhat relevant) were categorized as not relevant and scored as zero. The Content Validity Index (CVI) was then calculated at both the item level (I-CVI) and scale level (S-CVI) [49]. An item-level content validity index (I-CVI) of ≥ 0.78 was considered the minimum acceptable threshold, while a scale-level content validity index average (S-CVI/Ave) of ≥ 0.90 indicated acceptable overall content validity [48].

Furthermore, Gwet’s kappa coefficient was computed to assess the level of inter-rater agreement while accounting for chance agreement. Gwet’s kappa provides a more stable and reliable estimate of agreement; Cohen’s and Fleiss's kappa, in contrast, can yield negative values and tend to underestimate agreement in the case of imbalanced responses [50]. A kappa value < 0 indicates poor agreement; 0 to 0.20 indicates slight agreement; 0.21 to 0.40 indicates fair agreement; 0.41 to 0.60 indicates moderate agreement; 0.61 to 0.80 indicates substantial agreement; and 0.81 to 1 indicates almost perfect agreement [51,52].

### Face validity

The pre-final Amharic version of the PACIC-5As-ET tool was evaluated by twenty study participants at the University of Gondar Comprehensive Specialized Hospital. The participants were asked to review the questionnaire’s instructions and response categories, as well as the clarity and ease of understanding of each item. Any instruction, response format, or item identified as unclear by 20% or more of the participants was revised; those not meeting this threshold remained unchanged [48]. Both content and face validity confirmed the appropriateness and relevance of the tool among patients with T2D.

### Ceiling and floor effect

The ceiling effect occurs when a measurement instrument is not sensitive enough to detect higher levels of a variable, resulting in scores due to the clustering at the upper end. Conversely, the floor effect occurs when scores aggregate at the lower end of the scale, making it difficult to detect lower levels of a variable [53]. Both floor and ceiling effects indicate a limited or incomplete distribution of responses [54]. A floor or ceiling effect was identified if ≥ 15% of participants scored at the minimum (total score = 0) or maximum (total score = 100) levels, respectively [55–57]. In the present study, we identified ceiling and floor effects at the overall scale, subscale, and item levels.

### Reliability test

The reliability (internal consistency) of the instrument was assessed using Cronbach’s alpha coefficient and composite reliability (CR). Alpha measures the degree to which items produce consistency scores and should be interpreted as the proportion of measurement variance attributable to true score variance among individuals [58]. Internal consistency should range from 0 to 1, with values closer to 1 indicating higher internal consistency. A Cronbach’s alpha coefficient of ≥ 0.90 was interpreted as excellent, 0.80–0.90 as good, 0.70–0.80 as acceptable, 0.60–0.70 as questionable or needing improvement, 0.50–0.60 as poor, and < 0.50 as unacceptable [59,60].

Composite reliability was computed after factor analysis using standardized factor loadings of the constructs with the formula CR = (∑λ)² / [(∑λ)² + ∑(1-λ²)], where (∑λ)² represents the squared sum of factor loadings, and ∑(1-λ²) denotes the sum of error variance of the squared loadings [61,62]. According to Fornell and Larcker (1981), a CR value greater than 0.70 is considered acceptable, while values as low as 0.60 may also be deemed adequate [63].

### Inter-item and item-total correlation

The strength of relationships between items was evaluated using inter-item and item-total correlations. A two-tailed Pearson correlation was employed to examine the strength of association between items. An inter-item correlation of ≥ 0.30 was considered acceptable, and values below 0.30 indicated a weak correlation [64]. An item-total correlation (corrected item-total correlation) value of ≥ 0.20 was deemed acceptable, while a value below 0.20 was considered less acceptable [64,65].

### Test-retest reliability

Test-retest reliability reflects the consistency of scores obtained from the same respondents at two different times [66]. The test-retest reliability indicates the stability of the results over time [67]. A total of 40 participants with T2D participated in the test-retest assessment [68], which was conducted two weeks after the initial test [69,70]. Test-retest reliability was evaluated using the Intraclass Correlation Coefficient (ICC) based on a two-way mixed-effects model. ICC values less than 0.5 indicate poor reliability; 0.5–0.75 indicate moderate reliability; 0.75–0.90 indicate good reliability; and > 0.90 is interpreted as excellent test-retest reliability [71].

### Confirmatory factor analysis (CFA)

In this study, second-order (hierarchical) confirmatory factor analyses were conducted. Second-order factors should account for a significant portion of the variance in both the lower-order factors and the observed (manifest) variables, demonstrating their relevance and explanatory power across all levels of the model [62]. Prior to conducting the CFA, we checked the normality assumption using skewness and kurtosis. For normally distributed data, skewness and kurtosis values should generally fall within ±2, although kurtosis values up to ±7 may be acceptable [72,73].

In this confirmatory factor analysis, we evaluated the factor structures of PACIC-5As and its subscales, as originally proposed by Glasgow et al. (2005) [32], to determine whether the observed items adequately represented the underlying constructs and supported the proposed theoretical model [74].

Model fit and factor loadings were also assessed as part of this CFA. The model fit of the proposed PACIC-5As instrument and its constructs was evaluated using the Generalized Least Squares (GLS) method. In factor analysis, GLS can be used as the primary alternative fitting function to maximum likelihood, particularly when the observed variables are correlated and underlying assumptions of multivariate normality are violated [75,76]. GLS addresses the issue of estimating correlated errors, which is important for accurate model fitting and interpretation [77]. Similarly, GLS allows for more flexible assumptions about error variances and is a more powerful tool than other estimators for obtaining more efficient and unbiased estimates in the presence of heteroskedasticity [76–79].

To assess the model fit, various fit indices were evaluated. The absolute fit indices included the chi-square test (χ²), the chi-square test to degree of freedom ratio (χ²/df), the Root Mean Square Error of Approximation (RMSEA), the Root Mean Residual (RMR), the Standardized Root Mean Square Residual (SRMR), the Goodness of Fit Index (GFI), the Adjusted Goodness of Fit Index (AGFI), and the Parsimony Goodness of Fit Index (PGFI). The relative (incremental) fit indices, including the Comparative Fit Index (CFI), the Parsimony Comparative Fit Index (PCFI), the Incremental Fit Index (IFI), the Normed Fit Index (NFI), the Parsimony Normed Fit Index (PNFI), the Relative Fit Index (RFI), and the Tucker-Lewis Index (TLI), were used to assess model fitness.

Model fitness was evaluated using the recommended thresholds; however, the thresholds for fit indices can vary based on model complexity and research context [80]. An acceptable model fit indicated by the non-significant chi-square test (p-value > 0.05) or a ratio of chi-square to degree of freedom (χ²/df) ≤ 3, which is considered an acceptable fit [81]. RMSEA values less than 0.05 are considered an acceptable fit, and values up to 0.08 or 0.1 are also considered acceptable, while other studies suggest that a value close to zero indicates an acceptable fit [80]. For RMR and SRMR, a value ≤ 0.08 is deemed an acceptable fit, although other studies suggest that values closer to zero are considered acceptable [80]. For the other fit indices, such as GFI, AGFI, IFI, CFI, NFI, and PRATIO, values ≥ 0.90 are generally considered an acceptable fit; also, other studies suggest that values closer to 1 are considered acceptable [80,81]. In contrast, the smallest values are preferred for the Akaike Information Criterion (AIC) and Bayesian Information Criterion (BIC) [80,82]. There are no universal criteria or rules for the number of fit indices required to confirm good model fit. However, a model is generally considered to have an acceptable fit when at least two fit indices meet the recommended threshold values [83]. It is also possible for a model to adequately fit the data despite certain fit indices indicating a poor fit [81]. Likewise, standardized factor loading coefficients were evaluated at both the item and construct levels, with values greater than or equal to 0.40 considered acceptable [61,84].

Moreover, bootstrapping and modification indices were applied to enhance the accuracy and robustness of statistical estimates as well as to improve model fit. Bootstrapping is particularly useful when dealing with small sample sizes or non-normally distributed data [85]. Conversely, modification indices help reduce error terms and refine model fitness by adding covariances within a construct’s indicators or relationship paths between constructs, thereby adjusting the chi-square test value [78,85].

### Construct validity

After assessing the model fit, construct validity was evaluated. Construct validity refers to the degree to which a measurement tool accurately measures the theoretical construct it intends to measure [31]. In this study, both convergent and discriminant validity were assessed. Convergent validity was evaluated by calculating the average variance extracted (AVE) for the first-order model (between the subscales and observed variables), also known as the measurement model, and the second-order model (between the overall scale and subscales), also known as the structural model.

The AVE was computed using the formula: AVE = ∑λ² / [∑λ² + ∑(1-λ²)], where ∑λ² is the sum of the standardized squared loadings of the items, and ∑(1-λ²) is the sum of the error variance of the squared loading of the items [61,62]. According to Fornell and Larcker (1981) and other reporting guidelines, the AVE of the construct should be greater than or equal to 0.5 for observed variables [62,63]. However, if the composite reliability (CR) of the construct is above 0.6, an AVE as low as 0.4 is considered acceptable. Discriminant validity between each scale was evaluated using the AVEs values and the correlations between the constructs. Discriminant validity is considered high when the square root of the AVEs exceeds the inter-construct correlations [86,87].

### Data quality assurance

The tool was properly translated, culturally adapted, and validated in accordance with standardized guidelines [48]. Furthermore, we documented and reported all steps of the translation and validation process, including decisions made by the expert committee. Three data collectors with Master of Science (MSc) degrees in nursing and three MSc-level supervisors were recruited for the data collection process. All data collectors were recruited from the chronic care department within the hospital and had prior experience in data collection. However, to minimize potential social desirability bias, each data collector was recruited from a different chronic care unit (HIV, TB, and cancer care unit), excluding the diabetes care unit.

Data were collected using the Kobo Toolbox software. Prior to data collection, a four-day training session was conducted for the data collectors on the study objectives, data collection procedures, and the use of the KoboTool software. Before the main data collection began, 10 sample questionnaires were collected to identify and correct any errors in the questionnaire and to assess the functionality of the Kobo software.

During the data collection period, data collectors submitted their completed tasks to the principal investigator daily and reported any issues that required corrections to their supervisors by phone or in person. The principal investigator addressed any challenges or incidents that arose during the data collection period. Every day the supervisors monitored the daily activities of the data collectors and submitted a daily progress report to the principal investigator. The data collection process and reporting were completed within the planned timetable. All data were accurately recorded and documented, ensuring transparency and supporting the study’s reliability. The findings were reported according to the STROBE guideline for observational studies [88].

### Ethical consideration

This study was approved by the Institutional Research Ethics Review Committee (IRERC) of the University of Gondar College of Medicine and Health Sciences (CMHS) (IRERC/65/102025). All methods and procedures were carried out in accordance with the ethical principles outlined in the Declaration of Helsinki [89]. Permission and support letters were secured from the Amhara Public Health Institution (APHI). Additional authorization was also obtained from the chronic care center coordinator of each hospital. The study participant was informed about the purpose, methods, expected benefits, and risks of the study. Before data collection, written informed consent was obtained from each participant. Participants were informed that they had the right to decline participation in the study, and they had also been assured that refusal to participate would not affect their usual healthcare service in the hospital. Participants were informed that their confidentiality would be protected and that no personal information would be disclosed to third parties. To ensure confidentiality, codes were used to identify participants. For those who could not read or write, a thumbprint was used in place of the participant's signature.

## Results

### Translation and cultural adaptation

The PACIC-5A measurement tool was accurately translated by forward and backward translators. Subsequently, the expert committee thoroughly reviewed and evaluated each translation, reaching a consensus that the tool was relevant for measuring perceived quality of care. No items were removed from the original questionnaire.

Two forward translators, FT-1 and FT-2, independently translated the original English version of the PACIC-5As into Amharic. After completing their independent translations, the two translators met to discuss and synthesize their version into a single Amharic translation. They agreed that most items were accurately translated, with only minor grammatical errors. Items 4, 7, and 17 showed consistent translation by both translators. After correcting these grammatical errors, they produced a preliminary Amharic version called FT-12.

Next, FT-12 was translated back into English by two independent back translators (BT-1 and BT-2). Both translators completed their translations independently, after which they met to verify the items and content equivalence. The committee then reviewed the instructions, items, and response formats of all translations. They compared the back translations (BT-1 and BT-2) and forward translations (FT-1, FT-2, and FT-12) with each other and with the original English version.

During the panel discussion, BT-1 and BT-2 were thoroughly evaluated and compared to identify issues related to sentence structure, clarity, and meaning, with the goal of determining which version most accurately reflected the original intent of the tool. Items 2, 3, 4, 5, 6, 8, 9, 10, 12, 13, 16, 17, 21, 23, 24, 25, and 26 from BT-1 were selected as the most consistent with the original English version. In contrast, items 1, 7, 11, 14, 15, 18, 19, 20, and 22 were taken from BT-2. Minor grammatical corrections were made to the selected items. Finally, the committee confirmed the content equivalence of the tool and produced the final English version of the PACIC-5As tool (S1 Table), which aligns with the original version.

The committee also reviewed the Amharic versions (FT-1, FT-2, and FT-12) in comparison to the original English language version. They reviewed and corrected grammatical errors and selected culturally appropriate wording, meanings, and interpretations of the Amharic translations that aligned with the original English version. Finally, the committee established the initial conceptual, content, and semantic equivalence of the tool and approved the pre-final Amharic version of the PACIC-5As-ET tool for use in Ethiopia (S2**-**S3 Table).

The pre-final Amharic version (PACIC-5As-ET) was evaluated with twenty patients living with type 2 diabetes at the University of Gondar CSRH. The participants were asked to evaluate and provide feedback on the newly translated tool, focusing on its clarity, sentence construction, response format, meaning, and ease of understanding. The majority of participants (19/20) agreed that the instructions, response format, and items of the tool were clear and easy to understand. However, one participant (5%) commented that item 6, “Have you been guided to understand how the actions you have taken to care for yourself have contributed to your current condition?” was unclear, and another participant noted that item 10, “Have you been advised to go to a specific group or specialist who can provide support or assistance to improve or cope with your condition?” was unclear. Therefore, less than 20% of the participants reported the items were unclear; as a result, the items were not re-evaluated.

### Content validity

The content validity index was evaluated and rated by ten committee members. The content validity was computed at both the item and scale levels. The item-level content validity index (I-CVI) was ≥ 80 (80% to 100%), and the average or scale-level content validity index (S-CVI/Ave) was 97.69%; all were found to be acceptable. Moreover, the inter-rater agreement (Gwet’s kappa value) was 95% (95% CI: 0.91–0.99) with a p-value of < 0.001, indicating perfect agreement. The content validity assessment revealed that the measurement tool was relevant, valid, and accurately represented the intended construct.

### Face validity

In addition to the expert evaluation, the face validity of the newly translated and validated Amharic version of the PACIC-5As-ET measure was assessed by 20 study participants at the University of Gondar Comprehensive Specialized Hospital. We received valuable feedback from the participants regarding sentence construction, relevance, and clarity. Based on their input, almost all individual items were found to be correct and easily understood by the participants. Overall, the face validity assessment indicated that the measurement tool was clear, relevant, valid, and accepted by the majority of the study participants with T2D.

### Socio-demographic characteristics of the study participants

A total of 520 study participants were enrolled, of whom 517 were included, with a response rate of 99.4%. The mean age of the respondents was 54.34 (±11.51) years. More than half of the participants were male, 285 (55.1%), and 384 (74.5%) identified as Orthodox Christians, while 497 (96.1%) belonged to the Amhara ethnic group. The majority of participants, 410 (79.3%), resided in urban areas, and most respondents, 361 (69.8%), were married. Regarding educational status, the largest proportion, 161 (31.1%), had attained a college education or higher. Among occupations, the most common was housewives, comprising 114 (22.1%) individuals. Most participants, 389 (75.2%), had been diagnosed with diabetes for a period ranging from 6 months to 5 years (**Table 1**).

**Table 1:** Sociodemographic characteristics of patients with T2D attending in the Amhara region CSRHs, 2025 (n = 517).

Variables	Category	Frequency	Percentage (%)
Sex	Female	232	44.9
	Male	285	55.1
Age	25-44	101	19.5
	45-64	300	58.0
	≥65	116	22.4
Religion	Orthodox	385	74.5
	Muslim	120	23.2
	Catholic	2	0.4
	Protestant	10	1.9
Marital status	Married	361	69.8
	Divorced	38	7.4
	Separated	20	3.9
	Single	35	6.8
	Widowed	63	12.2
Educational status	College and above	161	31.1
	Secondary school	100	19.3
	Primary school	67	13.0
	Able to read and write	74	14.3
	Not able to read or write	115	22.2
Occupation	Government employee	85	16.4
	Private employee	48	9.3
	Unemployed	30	5.8
	Retired	80	15.5
	Merchant	87	16.8
	Housewife	114	22.1
	Farmer	56	10.8
	Daily laborer	17	3.3
Place of residence	Rural	107	20.7
	Urban	410	79.3
Ethnicity	Amhara	497	96.1
	Tigray	18	3.5
	Oromo	2	4
Diabetes duration	6 months to 5 years	389	75.2
	5 to 10 years	88	17.0
	>10 years	40	7.7

### Descriptive statistics of the PACIC-5As-ET measure

In this study, the overall mean summary score of PACIC-5As was 2.68 (± 0.62). The mean subscale scores were 2.79 (± 0.69) for Assess, 2.73 (± 0.71) for Advise, 2.63 (± 0.74) for Agree, 2.72 (± 0.69) for Assist, and 2.52 (± 0.76) for Arrange. Within the Assess subscale, item 21 had the highest mean score (3.57 ± 0.81), while item 11 had the lowest (2.25 ± 1.11). In the Advise subscale, item 6 had the highest mean (3.09 ± 0.79), whereas item 9 had the lowest (1.85 ± 1.05). For the Agree subscale, the highest score was recorded for item 3 (3.48 ± 0.88), and the lowest for item 25 (1.99 ± 1.26). In the Assist subscale, item 12 had the highest mean score (3.26 ± 0.97), while item 26 had the lowest (2.07 ± 1.16). In the Arrange subscale, the highest mean was observed for item 16 (3.21 ± 0.76) and the lowest for item 18 (2.02 ± 1.10) (**Table 2**).

**Table 2:** Mean and standard deviation of the PACIC-5As-ET measure among patients with T2D attending in the Amhara region CSRHs, 2025 (n = 517).

PACIC-5As scale, subscales and items	Minimum score	Maximum score	Mean	Standard Deviation (SD)
**Overall PACIC-5As-ET**			2.68	0.62
**Assess**			2.79	0.69
Item 1	1	5	2.14	1.24
Item 11	1	5	2.25	1.11
Item 15	1	5	3.18	0.78
Item 20	1	5	2.79	0.89
Item 21	1	5	3.57	0.81
**Advise**			2.73	0.71
Item 4	1	5	2.41	1.23
Item 6	1	5	3.09	0.79
Item 9	1	5	1.85	1.05
Item 19	1	5	3.08	1.01
Item 24	1	5	3.20	0.99
**Agree**			2.63	0.74
Item 10	1	5	2.82	0.97
Item 12	1	5	3.26	0.97
Item 13	1	5	2.42	1.08
Item 14	1	5	2.55	0.99
Item 26	1	5	2.07	1.16
**Assist**			2.72	0.69
Item 2	1	5	2.43	0.99
Item 3	1	5	3.48	0.88
Item 7	1	5	3.21	0.85
Item 8	1	5	2.48	1.08
Item 25	1	5	1.99	1.26
**Arrange**			2.52	0.76
Item 16	1	5	3.21	0.76
Item 17	1	5	2.36	1.13
Item 18	1	5	2.02	1.10
Item 22	1	5	2.74	0.89
Item 23	1	5	2.29	1.05

### Ceiling and floor effect

The overall PACIC-5As-ET scale did not demonstrate floor or ceiling effects; only 0.2% of participants achieved the minimum and maximum scores. Similarly, the subscales also showed no floor effects, ranging from 0.2% for Arrange to 0.8% for Assist, and no ceiling effects, ranging from 0.2% for Agree to 1.4% for Arrange. The results indicated that the measurement tool possesses sufficient sensitivity to detect variations at both the lower and upper ends of the score distribution, demonstrating good content validity and reflecting a better distribution of responses [57]. Although fewer items showed a floor effect, none exhibited a ceiling effect (all < 15%), implying that the measure is sensitive to detecting high levels of variation, but not low levels.

### Reliability assessment

#### Internal consistency of the PACIC-5As-ET and its constructs.

The Cronbach's alpha for the overall PACIC-5As-ET was 0.93, indicating excellent internal consistency, while the subscale alpha values were 0.74 for Assess, 0.72 for Advise, 0.76 for Agree, 0.71 for Assist, and 0.82 for Arrange, indicating acceptable reliability. The composite reliability of the overall PACIC-5As-ET was 0.93, demonstrating excellent internal consistency, while the subscales fell within a good to acceptable range: 0.76 for Assess, 0.73 for Advise, 0.79 for Agree, 0.75 for Assist, and 0.83 for Arrange. The subscale reliability tests also confirmed that the items were consistent and suitable for measuring the underlying constructs (**Table 3**).

**Table 3:** Cronbach’s alpha, composite reliability, average variance extracted, and test-retest reliability of the PACIC-5As-ET and its subscales among patients with T2D attending in the Amhara region CSRHs, 2025 (n = 517).

Constructs	Cronbach’s alpha (α)	CR	AVE	Test-retest reliability
				ICC with 95% CI
Assess	0.74 (0.71-0.78)	0.76	0.50	0.70 (0.52-0.82)
Advise	0.72 (0.68-0.76)	0.73	0.47	0.77 (0.64-0.87)
Agree	0.76 (0.73-0.80)	0.79	0.50	0.66 (0.46-0.80)
Assist	0.72 (0.68-0.75)	0.75	0.52	0.87 (0.79-0.92)
Arrange	0.82 (0.80-0.85)	0.83	0.59	0.86 (0.78-0.92)
PACIC-5As-ET	0.93 (0.92-0.94)	0.93	0.93	0.94 (0.91-0.96)

**Note:** AVE, average variance extracted; CR, composite reliability; ICC, intraclass correlation coefficient

### Inter-item and item-total correlation

The study demonstrated that the inter-item correlation ranged from 0.06 (between items 19 and 22) to 0.69 (between items 18 and 23). Based on expert recommendations, the weakly correlated items 19 and 22 were retained in the questionnaire. The corrected item-total correlation ranged from 0.49 to 0.69 for Assess, 0.31 to 0.66 for Advise, 0.41 to 0.72 for Agree, 0.39 to 0.66 for Assist, and 0.53 to 0.66 for Arrange. These correlations demonstrated that almost all items were reliable and suitable for measuring the underlying constructs. Additionally, the ‘Cronbach’s alpha if item deleted’ values for each item indicated good internal consistency, showing that no single item significantly affected the reliability of the scale (Table 4).

**Table 4:** Item-total statistics of the PACIC-5As-ET scales among patients with T2D attending in Amhara region CSRHs, 2025 (n = 517).

Constructs	Items	Scale Mean if Item Deleted	Scale Variance if Item Deleted	Corrected Item-Total Correlation	Squared Multiple Correlation	Cronbach's Alpha if Item Deleted
**Assess**	Item 1	64.82	216.22	0.65	0.62	0.926
	Item 11	64.70	221.49	0.56	0.56	0.928
	Item 15	63.78	226.95	0.59	0.44	0.927
	Item 20	64.16	222.24	0.69	0.55	0.926
	Item 21	63.39	228.64	0.49	0.45	0.929
**Advise**	Item 4	64.55	219.96	0.54	0.55	0.928
	Item 6	63.87	227.38	0.55	0.47	0.928
	Item 9	65.11	219.76	0.66	0.55	0.926
	Item 19	63.87	230.89	0.31	0.45	0.931
	Item 24	63.76	227.58	0.42	0.46	0.929
**Agree**	Item 2	64.14	221.68	0.64	0.56	0.926
	Item 3	63.70	228.38	0.41	0.41	0.930
	Item 7	64.54	218.24	0.69	0.54	0.925
	Item 8	64.41	222.47	0.61	0.45	0.927
	Item 25	64.88	215.41	0.72	0.64	0.925
**Assist**	Item 10	64.49	219.19	0.66	0.54	0.926
	Item 12	63.49	226.02	0.55	0.44	0.928
	Item 13	63.75	224.24	0.64	0.54	0.927
	Item 14	64.48	219.75	0.64	0.48	0.926
	Item 26	64.97	224.96	0.39	0.37	0.931
**Arrange**	Item 16	63.75	228.43	0.53	0.45	0.928
	Item 17	64.59	221.23	0.56	0.57	0.928
	Item 18	64.94	219.26	0.63	0.66	0.926
	Item 22	64.23	225.85	0.55	0.49	0.928
	Item 23	64.68	219.45	0.66	0.65	0.926

### Correlation between constructs

The strength of the association between constructs was computed using Pearson correlation analysis. All constructs demonstrated a strong, statistically significant correlation (p < 0.001, two-tailed). Among the subscales, the strongest correlation was found between *Assess and Agree* (r = 0.79), while the weakest correlation was between *Arrange and Advise* (r = 0.43). When comparing the correlations between the overall PACIC-5As-ET score and its subscales, the strongest correlation was observed with Agree (r = 0.92), and the weakest was with Advise (r = 0.79) (**Table 5**).

**Table 5:** Correlation between constructs among patients with T2D attending CSRHs in the Amhara region, 2025 (n = 517).

Constructs	Correlation					
	Assess	Advise	Agree	Assist	Arrange	Overall PACIC-5As-ET
Assess	1	0.57^**^	0.79^**^	0.73^**^	0.75^**^	0.89^**^
Advise	0.57^**^	1	0.68^**^	0.71^**^	0.43^**^	0.79^**^
Agree	0.79^**^	0.68^**^	1	0.76^**^	0.70^**^	0.92^**^
Assist	0.73^**^	0.71^**^	0.76^**^	1	0.62^**^	0.89^**^
Arrange	0.75^**^	0.43^**^	0.70^**^	0.62^**^	1	0.82^**^
Overall PACIC-5As-ET	0.89^**^	0.79^**^	0.92^**^	0.89^**^	0.82^**^	1

** Pearson correlation is significant at the 0.01 level (2-tailed)

### Test-retest reliability

The average test-retest value of the overall PACIC-5As-ET was 0.94, with subscale ICC values ranging from 0.66 for “Agree” to 0.87 for “Assist.” All results demonstrated moderate to excellent internal consistency, indicating the stability of the measurement tool over time (**Table 3**).

### Confirmatory factor analysis

A confirmatory factor analysis (CFA) was conducted to assess the model fit and the factor structure of the hypothesized model. Before conducting the CFA, the normality assumption was assessed using skewness and kurtosis. The univariate normality assessment revealed skewness values ranging from −0.31 to 1.07 and kurtosis values ranging from 1.10 to 0.16, suggesting a normal distribution. The multivariate normality assessment indicated that a skewness value of 58 suggested a departure from normality.

Although various fit indices were used to assess the model's fitness, the chi-square (χ²) statistic was 781.7 with a p-value of < 0.001, indicating that the model did not fit well. However, the χ²/df ratio was 2.79, which meets the recommended threshold of ≤ 3, suggesting an acceptable fit. The values of RMSEA and SRMR were 0.06 and 0.08, respectively, both of which satisfy the acceptable threshold of ≤ 0.08. The RMR was 0.12, not satisfying the recommended threshold of ≤ 0.08. The GFI and PRATIO were 0.89, close to the recommended threshold of ≥ 0.90 or near 1, indicating an acceptable fit. Furthermore, the AGFI and PGFI were 0.86 and 0.73, respectively; both values were close to 1 but slightly below the recommended threshold of ≥ 0.90. The IFI (0.57), NFI (0.30), PNFI (0.28), RFI (0.20), CFI (0.40), PCFI (0.35), and TLI (0.32) were all below the recommended threshold of ≥ 0.90. Additionally, the AIC value (860) was lower compared to those of the independence and null models. Overall, the absolute fit indices were generally within the recommended range, whereas incremental fit indices were low; therefore, support for the five-factor structure is limited and should be interpreted with caution (**Table 6**).

**Table 6:** Model fit indices of the hypothesized model among patients with T2D attending in the Amhara region CSRHs, 2025 (n = 517).

Absolute fit indices								Relative (Incremental) fit indices					Parsimonious fit indices	
CMIN (χ^2^)	P-value	CMINN/df	GFI	AGFI	RMSEA	RMR	SRMR	NFI	RFI	TLI	CFI	IFI	PGFI	PRATIO
781.7	< 0.001	2.79	0.89	0.86	0.06	0.12	0.08	0.30	0.20	0.32	0.40	0.57	0.73	0.89

**Note:** CMIN (χ²), chi-square; CMIN/df, chi-square to degree of freedom ratio; GFI, Goodness of Fit Index; AGFI, Adjusted Goodness of Fit Index; RMSEA, Root Mean Square Error of Approximation; RMR, Root Mean Residual; SRMR, Standardized Root Mean Residual; NFI, Normed Fit Index; RFI, Relative Fit index; TLI, Tucker Lewis Index; CFI, Comparative Fit Index; IFI, Incremental Fit Index; PGFI, Parsimony Goodness of Fit Index; PRATIO, Parsimony Ratio.

To enhance the model fit, error covariances such as e1-e10, e3-e21, e2-e4, and e1-e16 were adjusted based on modification indices. As a result, some of the fit indices improved: the χ^2^/df ratio decreased from 3.01 to 2.79, the GFI increased from 0.87 to 0.89, the AGFI improved from 0.85 to 0.86, and the RMSEA decreased from 0.062 to 0.06; the remaining values remained unchanged.

The standardized regression weights revealed that the factor loadings ranged from 0.34 (Q-19) to 0.84 (Q18, 23, and 25) for the measurement model and from 0.95 (Arrange) to 0.99 (Agree) for the structural model. In the “Assess scale,” the highest loading was 0.79 (Q1), and the lowest loading was 0.52 (Q21). In the “Advise scale,” the highest loading was 0.78 (Q9), and the lowest was 0.34 (Q19). In the “Agree scale,” the highest loading was 0.84 (Q 25), and the lowest loading was 0.43 (Q 3). In the “Assist scale,” the highest loading was 0.77 (Q 10), and the lowest loading was 0.48 (Q 26). In the “Arrange scale,” the highest loading was 0.84 (Q 18 and 23), and the lowest loading was 0.56 (Q 16).

**Fig 1:**
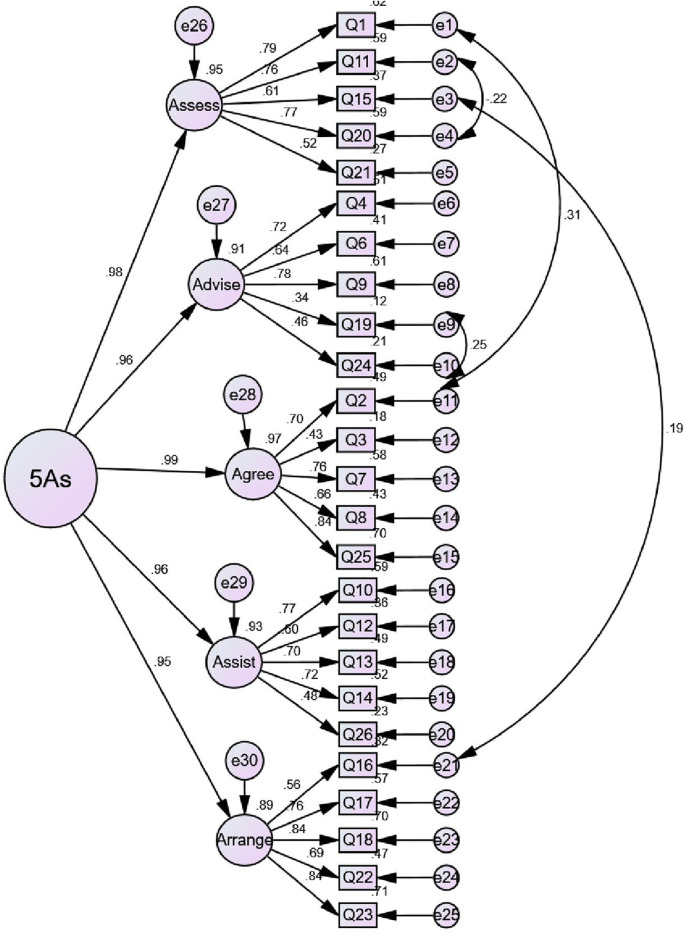
Second order confirmatory factor analysis among patients with T2D attending in Amhara region CSRHs, 2025 (n  = 517).

For the “Advise Scale” variables, item 19 of the newly validated and translated question (Question 19: “*Have you been told that being treated by other healthcare professionals like ophthalmologists, surgeons, and others helped you to improve your healthcare or treatment*?”) had a low loading (0.34). Before deleting the item, the expert committee evaluated its significance and decided to retain it in the questionnaire. Overall, almost all of the factor loadings exceeded the recommended threshold of 0.4, suggesting that the items represent their underlying constructs (Fig 1).

### Construct validity

Convergent and discriminant validities were assessed based on the Fornell-Larcker criterion. The convergent validity of the first-order constructs (measurement model) was 0.50 for Assess, 0.47 for Advise, 0.50 for Agree, 0.52 for Assist, and 0.59 for Arrange. All subscales showed adequate convergent validity. The convergent validity of the second-order construct (overall PACIC-5As) was 0.93, indicating strong convergent validity (**Table 3**). While the second-order construct demonstrated high discriminant validity, some of the first-order constructs exhibited lower discriminant validity (**Table 7**).

**Table 7:** Discriminant validity of the PACIC-5As-ET and its subscales among patients with T2D attending Amhara region CSRHs, 2025 (n = 517).

Constructs	Advise	Agree	Arrange	Assess	Assist	PACIC-5As-ET
Advise	**0.684**	0.717	0.506	0.628	0.717	0.825
Agree	0.717	**0.700**	0.647	0.762	0.768	0.890
Arrange	0.506	0.647	**0.766**	0.749	0.709	0.824
Assess	0.628	0.762	0.749	**0.709**	0.802	0.898
Assist	0.717	0.768	0.709	0.802	**0.722**	0.918

## Discussion

The patient assessment of chronic illness care (PACIC-5As) is a widely used tool to assess the perceived quality of care among individuals with chronic diseases, including key components associated with the behavioral change model [32]. In Ethiopia, the PACIC-5A tool has not yet been culturally adapted and validated, and there is limited research evaluating the quality of care from the patients’ perspectives [40]. Thus, there is a need to culturally adapt and utilize the PACIC-5As tool to assess the perceived quality of care among patients with T2D. Therefore, this study aimed to translate, culturally adapt, and evaluate the psychometric properties of the PACIC-5As questionnaire in Ethiopian adult patients with T2D.

The results demonstrated that the PACIC-5As measure was successfully translated, culturally adapted, and validated in Ethiopia. The tool was grammatically correct, clear, easy to understand, and acceptable among patients with T2D. In the current study, the I-CVI was ≥ 80.0% (80% to 100%), the S-CVI/Ave was 97.69%, and the average inter-rater agreement was 95.0%. These results are comparable to those of a study conducted in Saudi Arabia, where the S-CVI/Ave was 95.3%, and the average inter-rater agreement was 94.8% [34]. The findings are also consistent with those reported in an Iranian study, which showed an I-CVI value ≥ 87.0% (87% to 100%) and an S-CVI/Ave of 96.0% [35]. The content validity index of these studies exceeds the minimum threshold; it indicates that the measurement tool is relevant, valid, and accurately represents the intended construct.

The overall PACIC-5A-ET measure demonstrated no floor (0.2%) and ceiling effects (0.2%). The subscales also did not show a floor effect (0.2% to 0.8%) and a ceiling effect (0.2% to 1.4%). This aligns with a study reported in Thailand, where the overall summary score and subscales did not exhibit floor and ceiling effects, with all values below 15% [37]. In contrast, at the item level, the current study revealed a higher floor effect, ranging from 19.1% to 53.8%, and demonstrated no ceiling effect. These findings are comparable to a study reported in Greek, where the floor effect ranged from 3% to 31.5% [38], and in Thailand, the floor effect ranged from 16.6% to 53% [37]. The presence of a floor effect may indicate that some individuals misinterpreted the measurement tool or that the level of care received for that particular item was low [90]. This suggests that the measurement tool is less sensitive to detecting variation at the lower end of the score.

The internal consistency of the tool was assessed using Cronbach’s alpha and composite reliability, both of which demonstrated an excellent internal consistency of 0.93. The Cronbach’s alpha for the overall PACIC-5As-ET is comparable to the findings reported in the USA (0.97) [87], Saudi Arabia (0.90) [34], the Netherlands (0.91) [36], Thailand (0.91) [37], Greek (0.93) [38], and Iran (0.81) [35]. Moreover, the subscales of the current study showed good to acceptable results, with Cronbach’s alpha values ranging from 0.71 (Assist) to 0.82 (Arrange) and composite reliability values ranging from 0.73 (Advise) to 0.83 (Arrange), respectively. These values are comparable to those reported in Saudi Arabia, where subscale Cronbach’s alphas ranged from 0.70 (Assess) to 0.79 (Arrange) [34]. However, in Thailand, the Cronbach’s alpha for Advise was low (0.59), while the other values met the acceptable threshold: 0.68 (Assess), 0.74 (Agree), 0.75 (Assist), and 0.81 (Arrange) [37]. The lower Cronbach's alpha may be due to the small sample size in Thailand (151 participants) compared to the current study (517 participants). Conversely, the larger sample sizes in Greek (268) [38], Iran (306) [35], Saudi Arabia (557) [34], and the Netherlands (1941) [36] may contribute to achieving an acceptable threshold of internal consistency. Furthermore, the strong internal consistency indicated that the translation and validation process was effective and well understood by the participants, regardless of their language or residence.

The present study reported that inter-item correlation values ranged from 0.06 (between items 19 and 22) to 0.69 (between items 18 and 23), and item-total correlations ranged from 0.31 to 0.72. This finding is consistent with the study reported in Saudi Arabia, where item-total correlations ranged from 0.42 to 0.81 [34]. This indicated that most items are consistent in measuring the underlying construct.

The test-retest reliability of the PACIC-5As-ET tool was assessed two weeks after the initial evaluation. The test-retest ICC value for the overall PACIC-5As-ET was 0.94, indicating excellent reliability. The subscale ICC values ranged from 0.66 (Agree) to 0.87 (Assist), showing moderate to good internal consistency, respectively. The findings are higher than the study reported in Thailand, where the ICC value for the overall PACIC-5As was 0.59, and subscale ICC values ranged from 0.41 (Assess) to 0.60 (Arrange) [37]. This discrepancy may be attributed to differences in the test-retest duration; the Thailand study employed a longer test-retest interval (one month).

Confirmatory factor analysis was used to assess model fit and examine the factor structure of the hypothesized model (PACIC-5As-ET). The model demonstrated an acceptable fit, with χ²/df at 2.79, comparable to a study reported in Iran, where χ²/df was 2.54 [35]. The RMSEA value in the current study was 0.06, also comparable to the Iranian study (0.07) [35] and a study conducted in Greek (0.06) [38]. Additionally, the current study reported an SRMR value of 0.08, which is consistent with the Iranian study (0.08) [35]; however, the model fit was better than that of a Greek study (0.09) [38]. This can likely be attributed to the larger sample size in the current study, which probably enhanced the model fit [91,92]. The current study reported GFI (0.89) and AGFI (0.86), which are comparable to the GFI (0.85) and AGFI (0.81) found in the Iranian study [35]. Moreover, these results are consistent with the GFI (0.83) reported in the Greek study [38]. Moreover, the IFI for the current study was 0.57, while the Iranian study reported an IFI of 0.72 [35], both slightly below the recommended threshold of ≥ 0.9. The CFI in the current study was 0.40, indicating a poor fit compared to the CFI of 0.91 reported in Greek [38]. The lower value of the relative fit indices (CFI) may be due to a violation of the data distribution assumption. Furthermore, GLS can sometimes yield a high chi-square test value, negatively affecting the relative fit indices, even when the absolute fit indices indicate an acceptable fit [81].

Accordingly, construct validity was evaluated using standardized factor loadings and inter-construct correlations. The current study demonstrated factor loadings for Assess ranging from 0.52 (item-21) to 0.79 (item-1); Advise ranging from 0.34 (item-19) to 0.78 (item-9); Agree ranging from 0.43 (item-3) to 0.84 (item-25); Assist ranging from 0.48 (item-26) to 0.77 (item-10); and Arrange ranging from 0.56 (item-16) to 0.84 (items 18 & 23). All of these factor loadings were higher than those reported in the study reported in Iran, where Assess ranged from 0.10 (item-1) to 0.73 (item-21); Advise ranged from 0.09 (item-9) to 0.55 (item-21); Agree ranged from 0.20 (item-2) to 0.66 (item-7); Assist ranged from 0.27 (item-26) to 0.53 (item-14); and Arrange ranged from 0.17 (item-16) to 0.63 (item-23) [35]. These variations may be attributed to differences in sample size and the method of model estimation. The study reported in Iran had a relatively lower sample size than the current study, and an inadequate sample size may contribute to underestimating or lowering the factor loadings [92]. Furthermore, if the model is misspecified or the data are not normally distributed, there may be convergence issues and poor factor loadings [92,93].

The average variance extracted (AVE) for the overall PACIC-5As-ET was 0.93, indicating strong convergent validity, whereas the AVE values of the subscales ranged from 0.47 (Advise) to 0.59 (Arrange), indicating adequate convergent validity. In contrast, the structural model showed strong discriminant validity, particularly between the overall PACIC-5As-ET and its subscales. However, the subscales themselves showed less discriminant validity. This may be due to the inclusion of items with poor factor loadings, which contributed to reduced discriminant validity. Thus, in the current study, we excluded some items with factor loadings of ≤ 0.4 [94]. Moreover, high inter-construct correlations, those observed in the current study (≥ 0.68), can lead to low discriminant validity [95]. The other possible reason could be related to model specification, as the current study validated the preexisting PACIC-5As tool [32], which may include overlapping constructs that contributed to low discriminant validity [94].

### Implications of the study

The cross-cultural adaptation and psychometric evaluation of the PACIC-5As is important for assessing the perceived quality of care among patients with T2D. This study enables healthcare professionals and policymakers to identify gaps and enhance the quality of diabetes care. It also supports researchers in evaluating patient-centered care and provides insights to relevant stakeholders for informed decision-making. Moreover, this study promotes patient-centered care practices, enhances self-management support, and supports evidence-based decision-making to improve health outcomes for patients with T2D.

### Strengths and limitations of the study

In this study, we employed various statistical tests to assess the reliability and validity of the tool, including a test-retest assessment. We also utilized a maximum sample size (sample-to-parameter ratio), which may enhance the external validity of the study. However, the participants’ responses were clustered at the lower end of the scale, which may limit the tool's sensitivity to detect variations at that level. Although the incremental fit indices were low, the factorial validity should be interpreted with caution. In addition, the quality of care provided to diabetes patients may vary across healthcare settings, and studies conducted only in tertiary healthcare settings may limit generalizability to other healthcare settings, like primary and secondary healthcare settings.

## Conclusions

The Amharic version of the PACIC-5As-ET tool demonstrated excellent internal consistency and acceptable validity for assessing the perceptions of patients with T2D. It enables researchers to conduct research and supports healthcare providers in making evidence-based decisions to enhance the quality of diabetes care. In Ethiopia, Amharic is the national language spoken across various regions. However, to increase the applicability of the tool, large-scale studies should be conducted, including in primary, secondary, and tertiary healthcare settings.

## Supporting information

S1 Table English version of the PACIC-5As questionnaire. (DOCX)

S2 Table Amharic version of the PACIC-5As-ET questionnaire in Ethiopia. (DOCX)

S3 Table Summary table containing both English and Amharic version of the PACIC-5As questionnaire. (DOCX)

## References

[1] DeFronzo RA, Ferrannini E, Groop L, Henry RR, Herman WH, Holst JJ, et al. Type 2 diabetes mellitus. Nat Rev Dis Primers. 2015;1:15019. doi: 10.1038/nrdp.2015.19 27189025

[2] Sserwanja Q, Kamara K, Mutisya LM, Musaba MW, Ziaei S. Rural and urban correlates of stunting among under-five children in Sierra Leone: a 2019 nationwide cross-sectional survey. Nutr Metab Insights. 2021;14:11786388211047056. doi: 10.1177/11786388211047056 34616156 PMC8488416

[3] Khan MAB, et al. Epidemiology of Type 2 Diabetes - Global Burden of Disease and Forecasted Trends. J Epidemiol Glob Health. 2020;10(1):107–11. 32175717 10.2991/jegh.k.191028.001 PMC7310804

[4] Awad SF. Forecasting the type 2 diabetes mellitus epidemic and the role of key risk factors in Oman up to 2050: mathematical modeling analyses. J Diabetes Investig. 2021;12(7): 1162–74. 10.1111/jdi.13452 PMC8264408 33112504

[5] van Dieren S, Beulens JWJ, van der Schouw YT, Grobbee DE, Neal B. The global burden of diabetes and its complications: an emerging pandemic. Eur J Cardiovasc Prev Rehabil. 2010;17:S3-8. doi: 10.1097/01.hjr.0000368191.86614.5a 20489418

[6] Magliano DJ, Boyko EJ, I D F D A t e s. committee. IDF diabetes atlas. Brussels: International Diabetes Federation; 2021.

[7] Atlas ID. IDF diabetes atlas. International Diabetes Federation; 2019. http://www.idf.org/about-diabetes/facts-figures

[8] Sobngwi E. Diabetes in Africans. Part 1: epidemiology and clinical specificities. Diabetes Metabol. 2001;27(6):628–34. 11852370

[9] Manne-Goehler J, Atun R, Stokes A, Goehler A, Houinato D, Houehanou C, et al. Diabetes diagnosis and care in sub-Saharan Africa: pooled analysis of individual data from 12 countries. Lancet Diabetes Endocrinol. 2016;4(11):903–12. doi: 10.1016/S2213-8587(16)30181-4 27727123

[10] Zeru MA, Tesfa E, Mitiku AA, Seyoum A, Bokoro TA. Prevalence and risk factors of type-2 diabetes mellitus in Ethiopia: systematic review and meta-analysis. Sci Rep. 2021;11(1):21733. doi: 10.1038/s41598-021-01256-9 34741064 PMC8571297

[11] Marcovecchio ML, Lucantoni M, Chiarelli F. Role of chronic and acute hyperglycemia in the development of diabetes complications. Diabetes Technol Ther. 2011;13(3):389–94. doi: 10.1089/dia.2010.0146 21299400

[12] Cicek M, et al. Characterizing multimorbidity from type 2 diabetes: insights from clustering approaches. Endocrinol Metabol Clin North America. 2021;50(3):531–58. 10.1016/j.ecl.2021.05.012 PMC8383848 34399960

[13] Safieddine B, Sperlich S, Epping J, Lange K, Geyer S. Development of comorbidities in type 2 diabetes between 2005 and 2017 using German claims data. Sci Rep. 2021;11(1):11149. doi: 10.1038/s41598-021-90611-x 34045564 PMC8159920

[14] Heikkala E, Mikkola I, Jokelainen J, Timonen M, Hagnäs M. Multimorbidity and achievement of treatment goals among patients with type 2 diabetes: a primary care, real-world study. BMC Health Serv Res. 2021;21(1):964. doi: 10.1186/s12913-021-06989-x 34521389 PMC8442281

[15] Ferede YM, Erlandsson K, Gebrie MH, Beshah DT, Mohammed OY, Azagew AW, et al. Global prevalence of multimorbidity among people living with type 2 diabetes: a systematic review and meta-analysis. BMC Public Health. 2025;26(1):193. doi: 10.1186/s12889-025-25570-3 41382017 PMC12802173

[16] Epping-Jordan JE, Pruitt SD, Bengoa R, Wagner EH. Improving the quality of health care for chronic conditions. Qual Saf Health Care. 2004;13(4):299–305. doi: 10.1136/qhc.13.4.299 15289634 PMC1743863

[17] Clarke JL, Bourn S, Skoufalos A, Beck EH, Castillo DJ. An innovative approach to health care delivery for patients with chronic conditions. Popul Health Manag. 2017;20(1):23–30. doi: 10.1089/pop.2016.0076 27563751 PMC5278805

[18] Bergeson SC, Dean JD. A systems approach to patient-centered care. JAMA. 2006;296(23):2848–51. 17179462 10.1001/jama.296.23.2848

[19] Khunti K, Chudasama YV, Gregg EW, Kamkuemah M, Misra S, Suls J, et al. Diabetes and multiple long-term conditions: a review of our current global health challenge. Diabetes Care. 2023;46(12):2092–101. doi: 10.2337/dci23-0035 38011523 PMC10698221

[20] Regassa LD, Tola A. Magnitude and predictors of hospital admission, readmission, and length of stay among patients with type 2 diabetes at public hospitals of Eastern Ethiopia: a retrospective cohort study. BMC Endocr Disord. 2021;21(1):74. doi: 10.1186/s12902-021-00744-3 33866969 PMC8054433

[21] Skou ST. Multimorbidity. Nature Rev Disease Primers. 2022;8(1):48. 35835758 10.1038/s41572-022-00376-4 PMC7613517

[22] Evans R. Strengthening patient experience measurement and improvement. JAMA Health Forum. 2025;6(5):e251223–e251223. 10.1001/jamahealthforum.2025.1223 40445597

[23] Abid MH, et al. Patient-Centered Healthcare: From Patient Experience to Human Experience. Glob J Qual Saf Healthc. 2024;7(4):144–8. 39534234 10.36401/JQSH-24-X2 PMC11554389

[24] Marzban S, Najafi M, Agolli A, Ashrafi E. Impact of patient engagement on healthcare quality: a scoping review. J Patient Exp. 2022;9:23743735221125439. doi: 10.1177/23743735221125439 36134145 PMC9483965

[25] Cleary PD. The increasing importance of patient surveys: now that sound methods exist, patient surveys can facilitate improvement. British Medical Journal. 1999;:720–1. 10487981 10.1136/bmj.319.7212.720 PMC1116581

[26] Garratt AM, Schmidt L, Fitzpatrick R. Patient-assessed health outcome measures for diabetes: a structured review. Diabet Med. 2002;19(1):1–11. doi: 10.1046/j.1464-5491.2002.00650.x 11869297

[27] Schmittdiel J, Mosen DM, Glasgow RE, Hibbard J, Remmers C, Bellows J. Patient Assessment of Chronic Illness Care (PACIC) and improved patient-centered outcomes for chronic conditions. J Gen Intern Med. 2008;23(1):77–80. doi: 10.1007/s11606-007-0452-5 18030539 PMC2173922

[28] Wang MC, Bellows J. Quality of Life and Patient-Centered Outcomes. In: Daaleman TP, Helton MR, editors. Chronic Illness Care: Principles and Practice. Cham: Springer International Publishing; 2018. 95–107.

[29] McGlynn EA. Selecting common measures of quality and system performance. Med Care. 2003;41(1 Suppl):I39-47. doi: 10.1097/00005650-200301001-00005 12544815

[30] Wagner EH, Austin BT, Davis C, Hindmarsh M, Schaefer J, Bonomi A. Improving chronic illness care: translating evidence into action. Health Aff (Millwood). 2001;20(6):64–78. doi: 10.1377/hlthaff.20.6.64 11816692

[31] Arditi C, Iglesias K, Peytremann-Bridevaux I. The use of the Patient Assessment of Chronic Illness Care (PACIC) instrument in diabetes care: a systematic review and meta-analysis. Int J Qual Health Care. 2018;30(10):743–50. doi: 10.1093/intqhc/mzy091 29733366

[32] Glasgow RE, Whitesides H, Nelson CC, King DK. Use of the Patient Assessment of Chronic Illness Care (PACIC) with diabetic patients: relationship to patient characteristics, receipt of care, and self-management. Diabetes Care. 2005;28(11):2655–61. doi: 10.2337/diacare.28.11.2655 16249535

[33] Aung E, Ostini R, Dower J, Donald M, Coll JR, Williams GM, et al. Patient Assessment of Chronic Illness Care (PACIC) in Type 2 diabetes: a longitudinal study. Eval Health Prof. 2016;39(2):185–203. doi: 10.1177/0163278714556674 25380699

[34] Alharbi N, Alsubki N, Alotabi F, Alotabi M, Alhrabi N, de Lusgnian S, et al. Translation into arabic and validation of the patient assessment of care for chronic conditions questionnaire for diabetes. East Mediterr Health J. 2021;27(2):142–50. doi: 10.26719/emhj.20.136 33665798

[35] Maroufi S, Dehghankar L, Alizadeh A, Amerzadeh M, Motalebi SA. Transcultural adaptation and validation of Persian Version of Patient Assessment of Chronic Illness Care (PACIC-5As) Questionnaire in Iranian older patients with type 2 diabetes. BMC Health Serv Res. 2024;24(1):1073. doi: 10.1186/s12913-024-11557-0 39285400 PMC11404006

[36] Drewes HW, de Jong-van Til JT, Struijs JN, Baan CA, Tekle FB, Meijboom BR, et al. Measuring chronic care management experience of patients with diabetes: PACIC and PACIC+ validation. Int J Integr Care. 2012;12:e194. doi: 10.5334/ijic.862 23593054 PMC3601510

[37] Zeugfang D, Wisetborisut A, Angkurawaranon C, Aramrattana A, Wensing M, Szecsenyi J, et al. Translation and validation of the PACIC+ questionnaire: the Thai version. BMC Fam Pract. 2018;19(1):123. doi: 10.1186/s12875-018-0801-y 30025515 PMC6053714

[38] Malliarou M, Bakola E, Nikolentzos A, Sarafis P. Reliability and validity of the Greek translation of the patient assessment of chronic illness care + (PACIC-PLUS GR) survey. BMC Fam Pract. 2020;21(1):122. doi: 10.1186/s12875-020-01192-z 32586277 PMC7315532

[39] Nordin N, Hairon SM, Yaacob NM, Hamid AA, Isa SAM, Hassan N. Perceived quality of care among people with type 2 diabetes mellitus in the north east region of peninsular Malaysia. BMC Public Health. 2021;21(1):268. doi: 10.1186/s12889-021-10320-y 33568119 PMC7874640

[40] Birhanu F, et al. Patient-centered care and associated factors at public and private hospitals of Addis Ababa: patients’ perspective. Patient Relat Outcome Meas. 2021;12:107–16. 34045910 10.2147/PROM.S301771 PMC8144361

[41] Population EO, HC Commission. Summary and statistical report of the 2007 population and housing census: population size by age and sex. Federal Democratic Republic of Ethiopia, Population Census Commission; 2008.

[42] Bureau ANRH. Number of outpatients follow-up visits for type 2 diabetes. 2025.

[43] Gunawan J, Marzilli C, Aungsuroch Y. Establishing appropriate sample size for developing and validating a questionnaire in nursing research. Belitung Nurs J. 2021;7(5):356–60. doi: 10.33546/bnj.1927 37496511 PMC10367972

[44] Vrijhoef HJM, Berbee R, Wagner EH, Steuten LMG. Quality of integrated chronic care measured by patient survey: identification, selection and application of most appropriate instruments. Health Expect. 2009;12(4):417–29. doi: 10.1111/j.1369-7625.2009.00557.x 19709315 PMC5060503

[45] Glasgow RE, Wagner EH, Schaefer J, Mahoney LD, Reid RJ, Greene SM. Development and validation of the Patient Assessment of Chronic Illness Care (PACIC). Med Care. 2005;43(5):436–44. doi: 10.1097/01.mlr.0000160375.47920.8c 15838407

[46] Whitlock EP, Orleans CT, Pender N, Allan J. Evaluating primary care behavioral counseling interventions: an evidence-based approach. Am J Prev Med. 2002;22(4):267–84. doi: 10.1016/s0749-3797(02)00415-4 11988383

[47] Aghili R, Valojerdi AE, Farshchi A, Khamseh ME. Type 2 diabetes: patient assessment of chronic illness care. J Diabetes Metab Disord. 2021;20(1):7–13. doi: 10.1007/s40200-020-00540-1 34178820 PMC8212328

[48] Sousa VD, Rojjanasrirat W. Translation, adaptation and validation of instruments or scales for use in cross-cultural health care research: a clear and user-friendly guideline. J Eval Clin Pract. 2011;17(2):268–74. doi: 10.1111/j.1365-2753.2010.01434.x 20874835

[49] Polit DF, Beck CT. The content validity index: are you sure you know what’s being reported? Critique and recommendations. Res Nurs Health. 2006;29(5):489–97. doi: 10.1002/nur.20147 16977646

[50] Wongpakaran N, Wongpakaran T, Wedding D, Gwet KL. A comparison of Cohen’s Kappa and Gwet’s AC1 when calculating inter-rater reliability coefficients: a study conducted with personality disorder samples. BMC Med Res Methodol. 2013;13:61. doi: 10.1186/1471-2288-13-61 23627889 PMC3643869

[51] Roldán-Nofuentes JA, Regad SB. Estimation of the average kappa coefficient of a binary diagnostic test in the presence of partial verification. Mathematics. 2021;9(14):1694. doi: 10.3390/math9141694

[52] Landis JR, Koch GG. The measurement of observer agreement for categorical data. Biometrics. 1977;33(1):159–74. doi: 10.2307/2529310 843571

[53] Garin O. Ceiling effect. In: Maggino F, editor. Encyclopedia of quality of life and well-being research. Cham: Springer International Publishing; 2023. 704–6.

[54] Stucki G, Liang MH, Stucki S, Katz JN, Lew RA. Application of statistical graphics to facilitate selection of health status measures for clinical practice and evaluative research. Clin Rheumatol. 1999;18(2):101–5. doi: 10.1007/s100670050065 10357113

[55] Parviniannasab AM, Faramarzian Z, Hosseini SA, Hamidizadeh S, Bijani M. The effect of social support, diabetes management self-efficacy, and diabetes distress on resilience among patients with type 2 diabetes: a moderated mediation analysis. BMC Public Health. 2024;24(1):477. doi: 10.1186/s12889-024-18022-x 38360647 PMC10868118

[56] Berhe KK, Mselle LT, Gebru HB. Psychometric evaluation of the problem areas in diabetes (PAID) scale among people with type 2 diabetes in Ethiopia: a tool validation study. BMC Res Notes. 2025;18(1):154. doi: 10.1186/s13104-025-07238-8 40205624 PMC11983946

[57] Terwee CB, Bot SDM, de Boer MR, van der Windt DAWM, Knol DL, Dekker J, et al. Quality criteria were proposed for measurement properties of health status questionnaires. J Clin Epidemiol. 2007;60(1):34–42. doi: 10.1016/j.jclinepi.2006.03.012 17161752

[58] Forero CG. Cronbach’s alpha. In: Maggino F, editor. Encyclopedia of quality of life and well-being research. Cham: Springer International Publishing; 2023. 1505–7.

[59] Taber KS. The use of Cronbach’s alpha when developing and reporting research instruments in science education. Res Sci Edu. 2018;48(6):1273–96.

[60] Saidi SS, Siew NM. Investigating the validity and reliability of survey attitude towards statistics instrument among rural secondary school students. Int J Educ Methodol. 2019;5(4):651–61. doi: 10.12973/ijem.5.4.651

[61] Cheung GW, Cooper-Thomas HD, Lau RS, Wang LC. Reporting reliability, convergent and discriminant validity with structural equation modeling: a review and best-practice recommendations. Asia Pac J Manag. 2023;41(2):745–83. doi: 10.1007/s10490-023-09871-y

[62] Credé M, Harms PD. 25 years of higher-order confirmatory factor analysis in the organizational sciences: a critical review and development of reporting recommendations. J Organiz Behav. 2015;36(6):845–72. doi: 10.1002/job.2008

[63] Fornell C, Larcker DF. Evaluating structural equation models with unobservable variables and measurement error. J Market Res. 1981;18(1):39. doi: 10.2307/3151312

[64] Boateng GO, et al. Best practices for developing and validating scales for health, social, and behavioral research: a primer. Front Public Health. 2018;6:149. 29942800 10.3389/fpubh.2018.00149 PMC6004510

[65] Nunnally J, Bernstein I. Psychometric theory. 3rd ed. New York: MacGraw-Hill; 1994.

[66] Nguyen R, Brooks M, Bruno R, Peacock A. Behavioral measures of state impulsivity and their psychometric properties: a systematic review. Personal Indiv Diff. 2018;135:67–79. doi: 10.1016/j.paid.2018.06.040

[67] Vilagut G. Test-retest reliability. Encyclopedia of quality of life and well-being research. Springer; 2024. 7180–4.

[68] Kennedy I. Sample size determination in test-retest and cronbach alpha reliability estimates. British J Contemp Edu. 2022;2(1):17–29. doi: 10.52589/bjce-fy266hk9

[69] Park MS, Kang KJ, Jang SJ, Lee JY, Chang SJ. Evaluating test-retest reliability in patient-reported outcome measures for older people: a systematic review. Int J Nurs Stud. 2018;79:58–69. doi: 10.1016/j.ijnurstu.2017.11.003 29178977

[70] Matusova M, Maracek M, Pavelka J, Ng K, Medina C, Tylsarova N, et al. Measuring screen time among adolescents: test-retest reliability of HBSC questionnaire items across two countries. BMC Public Health. 2025;26(1):290. doi: 10.1186/s12889-025-25950-9 41413499 PMC12829222

[71] Koo TK, Li MY. A guideline of selecting and reporting intraclass correlation coefficients for reliability research. J Chiropr Med. 2016;15(2):155–63. doi: 10.1016/j.jcm.2016.02.012 27330520 PMC4913118

[72] Kim H-Y. Statistical notes for clinical researchers: assessing normal distribution (2) using skewness and kurtosis. Restor Dent Endod. 2013;38(1):52–4. doi: 10.5395/rde.2013.38.1.52 23495371 PMC3591587

[73] Garson GD. Testing statistical assumptions. Asheboro, NC: Statistical Associates Publishing; 2012.

[74] Hurley AE, Scandura TA, Schriesheim CA, Brannick MT, Seers A, Vandenberg RJ, et al. Exploratory and confirmatory factor analysis: guidelines, issues, and alternatives. J Organiz Behav. 1997;18(6):667–83. doi: 10.1002/(sici)1099-1379(199711)18:6<667::aid-job874>3.0.co;2-t

[75] Reyes-Carreto R, Godinez-Jaimes F, Guzmán-Martínez M. The basics of structural equations in medicine and health sciences. In: Recent advances in medical statistics. IntechOpen; 2022.

[76] Brown JD. Advanced statistics for the behavioral sciences. New York: Springer; 2018. https://doi.org/10

[77] Deng L, Yang M, Marcoulides KM. Structural equation modeling with many variables: a systematic review of issues and developments. Front Psychol. 2018;9:580. 29755388 10.3389/fpsyg.2018.00580 PMC5932371

[78] Wooldridge JM. Introductory econometrics. 5th ed. Cengage Learning; 2013.

[79] Korhonen P, Nordhausen K, Taskinen S. A review of generalized linear latent variable models and related computational approaches. WIREs Computational Stats. 2024;16(6). doi: 10.1002/wics.70005

[80] S S, Mohanasundaram T. Fit indices in structural equation modeling and confirmatory factor analysis: reporting guidelines. Asian J Econ Busin Acc. 2024;24(7):561–77. doi: 10.9734/ajeba/2024/v24i71430

[81] Schermelleh-Engel K, Moosbrugger H, Müller H. Evaluating the fit of structural equation models: Tests of significance and descriptive goodness-of-fit measures. Methods of psychological research online. 2003;8(2):23–74.

[82] Joreskog K, Sorbom D. Structural equation modelling: guidelines for determining model fit. NY: University Press of America; 1993.

[83] Hu L, Bentler PM. Cutoff criteria for fit indexes in covariance structure analysis: conventional criteria versus new alternatives. Struct Equ Model: A Multidiscipl J. 1999;6(1):1–55. doi: 10.1080/10705519909540118

[84] Boateng GO, Neilands TB, Frongillo EA, Melgar-Quiñonez HR, Young SL. Best practices for developing and validating scales for health, social, and behavioral research: a primer. Front Public Health. 2018;6:149. doi: 10.3389/fpubh.2018.00149 29942800 PMC6004510

[85] Byrne B, St C. Structural equation modeling with AMOS. 2022.

[86] Henseler J, Ringle CM, Sarstedt M. A new criterion for assessing discriminant validity in variance-based structural equation modeling. J of the Acad Mark Sci. 2014;43(1):115–35. doi: 10.1007/s11747-014-0403-8

[87] Rönkkö M, Cho E. An updated guideline for assessing discriminant validity. Organizat Res Methods. 2020;25(1):6–14. doi: 10.1177/1094428120968614

[88] von Elm E, Altman DG, Egger M, Pocock SJ, Gøtzsche PC, Vandenbroucke JP, et al. Strengthening the Reporting of Observational Studies in Epidemiology (STROBE) statement: guidelines for reporting observational studies. BMJ. 2007;335(7624):806–8. doi: 10.1136/bmj.39335.541782.AD 17947786 PMC2034723

[89] Association WM. Declaration of Helsinki: medical research involving human participants. World Medical Association; 2024.

[90] Salman AA. What are the priming and ceiling effects of one experience measure on another?. J Patient Exp. 2020;7(6):1755–9. 33457640 10.1177/2374373520951670 PMC7786675

[91] Indices AF. Some clarifications and recommendations on fit indices. 2010.

[92] Jackson DL, Voth J, Frey MP. A Note on sample size and solution propriety for confirmatory factor analytic models. Struct Equ Model: A Multidiscip J. 2013;20(1):86–97. doi: 10.1080/10705511.2013.742388

[93] Olsson UH, Foss T, Troye SV, Howell RD. The Performance of ML, GLS, and WLS Estimation in Structural Equation Modeling Under Conditions of Misspecification and Nonnormality. Structural Equation Modeling: A Multidisciplinary Journal. 2000;7(4):557–95. doi: 10.1207/s15328007sem0704_3

[94] Brown TA. Confirmatory factor analysis for applied research. Guilford Publications; 2015.

[95] Fornell C, Larcker DF. Structural Equation Models with Unobservable Variables and Measurement Error: Algebra and Statistics. J Market Res. 1981;18(3):382. doi: 10.2307/3150980

