# Supplementary material for: Translation, cultural adaptation, and psychometric evaluation of the Patient Assessment Chronic Illness Care tool in Ethiopia (PACIC-5As-ET) for patients with type 2 diabetes
Source: PLoS One. 2026 Jun 11;21(6):e0329197. doi: 10.1371/journal.pone.0329197 (PMC13258013; doi:10.1371/journal.pone.0329197)
Supplement: S1 Table — (DOCX) [file pone.0329197.s001.docx]

**S1 Table: English version of the PACIC-5As** questionnaire

**Instruction:** The following questions ask about your experience or perception of managing your diabetes over the past six months.

When you experience a chronic disease, it can be difficult to remain healthy. We want to learn from you about the healthcare services provided to you by the healthcare team. Such services might include the care your doctors or nurses provided. Your responses will be kept confidential and will not be shared with anyone else.

Over the past six months, when you have been receiving medical services for type 2 diabetes:

| **No.** | **Questions** | **Almost Never**  **(1)** | **It is not generally (2)** | **Sometimes (3)** | **Most of the time (4)** | **Almost Always (5)** |
| --- | --- | --- | --- | --- | --- | --- |
|  | I was asked to share my opinion when we prepared a treatment plan. | 1 | 2 | 3 | 4 | 5 |
|  | Have you ever been informed about and encouraged to be aware of and consider different treatment options? | 1 | 2 | 3 | 4 | 5 |
|  | Have you ever been informed to report any side effects or outcomes you experience while taking the medication? | 1 | 2 | 3 | 4 | 5 |
|  | Have you ever provided a written list of things to do to improve your health? | 1 | 2 | 3 | 4 | 5 |
|  | Do you feel satisfied that you have received appropriate follow-up and support? | 1 | 2 | 3 | 4 | 5 |
|  | Have you been guided to understand how the actions you have taken to care for yourself have contributed to your current condition? | 1 | 2 | 3 | 4 | 5 |
|  | Have you been asked to explain the goals of your self-care practices? | 1 | 2 | 3 | 4 | 5 |
|  | Have you ever been assisted in setting specific goals to improve your diet and physical activity? | 1 | 2 | 3 | 4 | 5 |
|  | Have you ever been provided with a copy of your treatment plan? | 1 | 2 | 3 | 4 | 5 |
|  | Have you been advised to go to a specific group or specialist who can provide support or assistance to improve or cope with your condition? | 1 | 2 | 3 | 4 | 5 |
|  | Have you been asked to explain your thoughts about your health experience in an interview or survey? | 1 | 2 | 3 | 4 | 5 |
|  | Can you confidently say that the doctor or nurse takes your values and traditions into account when providing care? | 1 | 2 | 3 | 4 | 5 |
|  | Do you believe that support has been provided to develop a healthcare plan that you can implement in your daily life? | 1 | 2 | 3 | 4 | 5 |
|  | Do you think you have been advised to prepare a plan ahead of time so you can take care of yourself, even in challenging situations? | 1 | 2 | 3 | 4 | 5 |
|  | Have you been asked how this chronic disease affects your life? | 1 | 2 | 3 | 4 | 5 |
|  | Have you ever been asked about the state of your health after receiving treatment? | 1 | 2 | 3 | 4 | 5 |
|  | Do you think that you have been encouraged to participate in programs within the community that can help you? | 1 | 2 | 3 | 4 | 5 |
|  | Have you ever been informed to seek out dietitians, health educators, or counselors? | 1 | 2 | 3 | 4 | 5 |
|  | Have you been told that being treated by other healthcare professionals like ophthalmologists, surgeons, and others helped you to improve your healthcare or treatment? | 1 | 2 | 3 | 4 | 5 |
|  | Have you been asked how the care you received from other doctors helped you? | 1 | 2 | 3 | 4 | 5 |
|  | Have you ever been asked about what topics you would like to discuss with your doctor during your appointments? | 1 | 2 | 3 | 4 | 5 |
|  | Have you been asked if you have faced any problems related to self-care in your workplace, with your families, or with social conditions? | 1 | 2 | 3 | 4 | 5 |
|  | Have you been supported to develop a plan to receive support from your friends, family, and community? | 1 | 2 | 3 | 4 | 5 |
|  | Have you ever been informed about how important certain self-care activities (e.g., physical exercise) are for your health? | 1 | 2 | 3 | 4 | 5 |
|  | Have you ever set a goal with your healthcare team to manage your health? | 1 | 2 | 3 | 4 | 5 |
|  | Have you been provided a follow-up record or book to monitor your progress or improvements in your health? | 1 | 2 | 3 | 4 | 5 |

**Scoring methods of PACIC-5As**

| For the overall summary score | Average of items 1-4 and 6-26 (excluding item 5) |
| --- | --- |
| Assess | Average of items 1, 11, 15, 20, and 21 |
| Advice | Average of items 4, 6, 9, 19, and 24 |
| Agree | Average of items 2, 3, 7, 8, and 25 |
| Assist | Average of items 10, 12, 13, 14, and 26 |
| Arrange | Average of items 16, 17, 18, 22, and 23 |
