# Supplementary material for: Translation, cultural adaptation, and psychometric evaluation of the Patient Assessment Chronic Illness Care tool in Ethiopia (PACIC-5As-ET) for patients with type 2 diabetes
Source: PLoS One. 2026 Jun 11;21(6):e0329197. doi: 10.1371/journal.pone.0329197 (PMC13258013; doi:10.1371/journal.pone.0329197)
Supplement: S2 Table — (DOCX) [file pone.0329197.s002.docx]

**S2 Table:** Amharic version of the PACIC-5As-ET questionnaire in Ethiopia

**መመሪያ** ፡ ከዚህ በታች ያሉት ጥያቄዎች ባለፉት ስድስት ወራት ውስጥ ስላገኙት የህክምና እንክብካቤ ልምድ ወይም አስተሳሰብ የሚመለከት መጠይቅ ነው።

| ቆየት ያለ ህመም ሲያጋጥምዎ ጤናማ ሆኖ መቆየት አስቸጋሪ ሊሆን ይችላል. ከእርስዎ የጤና ተንከባካቢ ቡድን ስለ እርስዎ ሁኔታ ስላለው የእርዳታ አይነት መማር እንፈልጋለን። ይህ ምናልባት መደበኛ ህክምና የሚሰጥወትን ሀኪም ወይም ነርስን ሊያካትት ይችላል፡፡ መልሶችዎ በሚስጥር ይጠበቃሉ ለሌላም ለማንም አይነገርም። |
| --- |

**ላለፉት ስድስት ወራት ለስኳር ህመም የህክምና አገልግሎት ሲወስዱ፦**

| **ተ.ቁ** | **መጠይቆች** | **በጭራሽ ማለት ይቻላል**  **(1)** | **በአጠቃላይ አይደለም (2)** | **አንዳንዴ (3)** | **አብዛኛውን ጊዜ (4)** | **ሁልጊዜ ማለት ይቻላል (5)** |
| --- | --- | --- | --- | --- | --- | --- |
|  | የህክምና እቅድ በሚዘጋጅበት ጊዜ ሀሳብዎን እንዲያጋሩ ተጠይቀው ያውቃሉ? | 1 | 2 | 3 | 4 | 5 |
|  | ስለተለያዩ የህክምና አማራጮች እንዲያዉቁና እንዲያስቡቧቸዉ ተደርጎ ያዉቃል? | 1 | 2 | 3 | 4 | 5 |
|  | መድሃኒቱን በሚወስዱበት ወቅት ሰላጋጠመዎት የጎንዮሽ ጉዳት ወይም ዉጤት ተጠይቀው ያውቃሉ? | 1 | 2 | 3 | 4 | 5 |
|  | ጤንነትዎን ለማሻሻል ማድረግ የሚገባዎትን ነገር በጽሁፍ ተዘርዝረው ተሰጥተዎት ያውቃል? | 1 | 2 | 3 | 4 | 5 |
|  | የተሰጠኝ አገልግሎት የተሟላ ስለሆነ ረክቻለሁ ብለዉ ያስባሉ? | 1 | 2 | 3 | 4 | 5 |
|  | እራስዎትን ለመንከባከብ ያደረጓቸዉ ነገሮች አሁን ላሉበት ሁኔታ እንዴት እንዳገዙ እንዲገነዘቡ ተደርጎ ያወቃል? | 1 | 2 | 3 | 4 | 5 |
|  | ከህመምዎ ጋር በተያያዘ የሚያደርጓቸው እንክብካቤ አላማቸው ምን እደሆነ ተጠይቀው ያውቃሉሁ? | 1 | 2 | 3 | 4 | 5 |
|  | አመጋገብዎትንም ሆነ የአካል ብቃት እንቅስቃሴዎን ለማሻሽል የሚረዳዎትን ዝርዝር አላማዎችን እንዲያወጡ እገዛ ተደርጎልዎት ያዉቃል? | 1 | 2 | 3 | 4 | 5 |
|  | የህክምና እቅድዎ ቅጅ ተሰጥትዎት ያዉቃል? | 1 | 2 | 3 | 4 | 5 |
|  | ጤናዎት በደንብ እንዲሻሻል ልዩ ድጋፍ ወይም እርዳታ ወደ ሚያደርግልዎት ባለሙያ እንድሄዱ ተደርጓል? | 1 | 2 | 3 | 4 | 5 |
|  | ስለጤና ልምድዎ በቃለ ምልልስም ሆነ በዳሰሳ ጥናት ሀሳብዎትን እንዲገልፁ ተደርጎ ያዉቃል? | 1 | 2 | 3 | 4 | 5 |
|  | ዶክተሮቹም ሆነ ነርሶቹ ህክምና አገልግሎት ሲሰጡ ያለዎትን ባህልና ወጎች ግምት ውስጥ ያስገባ ነዉ ብለዉ በእርግጠኝነት መናገር ይችላሉ? | 1 | 2 | 3 | 4 | 5 |
|  | በእለት ተእለት ህይዎትዎ ማከናወን የሚችሉትን የህክምና እቅድ እንዲያወጡ እገዛ ተደርጎልዎታል? | 1 | 2 | 3 | 4 | 5 |
|  | ሙያተኞች በአስቸጋሪ ሁኔታዎችም ቢሆን ራስዎን ለማስታመም እንዲችሉ አስቀድመዉ እቅድ እንዲዘጋጅ ረድተዉኛል ብለዉ ያስባሉ? | 1 | 2 | 3 | 4 | 5 |
|  | ያልብዎት የጤና ችግር ህይዎትዎ ላይ ስላደረሰው ተጽኖ ተጠይቀዉ ያዉቃሉ? | 1 | 2 | 3 | 4 | 5 |
|  | ህክምና ከተደረገልዎት በኋላ ጤናዎ በምን ሁኔታ ላይ እንዳለ ለማወቅ ተጠይቀዉ ያዉቃሉ | 1 | 2 | 3 | 4 | 5 |
|  | በማኅበረሰቡ ውስጥ ሊረዱኝ በሚችሉ ፕሮግራሞች ላይ እንድገኝ ተበረታትቻለሁ ብለው ያስባሉ? | 1 | 2 | 3 | 4 | 5 |
|  | የስነምግብ ባለሙያ፣ የጤና አስተማሪ ወይም አማካሪ እንዲያገኙ ተልከው ያዉቃሉ? | 1 | 2 | 3 | 4 | 5 |
|  | በሌሎች የህክምና ባለሙያዎች ለምሳሌ በዓይን ሀኪም፣ በቀዶ ጥገና ሀኪሞች እና በመሳሰሉት መታየት እንዴት ህክምናዎን እንደሚያግዝ ተነግሮዎት ያዉቃል? | 1 | 2 | 3 | 4 | 5 |
|  | ከሌሎች ሀኪሞች ጋር ያለዎት ክትትል እንዴት እንደሆነ ተጠይቀዉ ያዉቃሉ? | 1 | 2 | 3 | 4 | 5 |
|  | በክትትልዎ ስአት ስለምን ማዉራት እንድሚፈልጉ ተጠይቀዉ ያዉቃሉ? | 1 | 2 | 3 | 4 | 5 |
|  | ከስራዎ፤ ከቤተሰብዎ ወይም ከማህበራዊ ሁኔታ ጋር በተያያዘ ራስዎን ለመንከባከብ ያጋጠመዎት ችግር መኖሩን ወይም አለመኖሩን ተጠይቀዉ ያዉቃሉ? | 1 | 2 | 3 | 4 | 5 |
|  | ከጓደኞዎ፣ ከቤተሰብዎ እንዲሁም ከማህበረሰቡ እርዳታ ለማግኘት የሚያስችልዎትን እቅድ ለማዘጋጀት እገዛ ተደርጎልዎት ያዉቃል? | 1 | 2 | 3 | 4 | 5 |
|  | እራስዎትን ለመንከባከብ የሚያደርጓቸዉ ነገሮች (ለምሳሌ የአካል ብቃት እንቅስቃሴ) ለጤናዎ ምን ያህል አስፈላጊ እንደሆኑ ተነግሮዎት ያዉቃል? | 1 | 2 | 3 | 4 | 5 |
|  | ከጤና ሙያተኞች ጋር በመሆን ጤናዎን ለመጠበቅ የሚያስችልዎትን እቅድ አዉጥተዉ ያዉቃሉ? | 1 | 2 | 3 | 4 | 5 |
|  | ያለዎትን የጤና መሻሻል ለመመዝገብ የሚረዳዎት መዝገብ (ደብተር) ተስጥቶዎት ያውቃል? | 1 | 2 | 3 | 4 | 5 |

**የህክምና እንክብካቤ የነጥብ አሰጣጥ ዘዴዎች (scoring methods of the PACIC-5As-ET tool)**

| አጠቃላይ የህክምና እንክብካቤ ጥያቄ (overall summary score of PACIC-5As) | ጥያቄ ቁጥር ከ 1-4 እና ከ 6-26 ( ጥያቄ ቁጥር “5” አያካትትም) አማካኝ ውጤት |
| --- | --- |
| መገምገም (Assess) | የጥያቄ ቁጥር 1, 11, 15, 20, እና 21 አማካኝ ውጤት |
| ማማከር (Advice) | የጥያቄ ቁጥር 4, 6, 9, 19, እና 24 አማካኝ ውጤት |
| መስማማት (Agree) | የጥያቄ ቁጥር 2, 3, 7, 8, እና 25 አማካኝ ውጤት |
| ማገዝ (Assist) | የጥያቄ ቁጥር 10, 12, 13, 14, እና 26 አማካኝ ውጤት |
| ማዘጋጀት (Arrange) | የጥያቄ ቁጥር 16, 17, 18, 22 እና 23 አማካኝ ውጤት |
