# Supplementary material for: Translation, cultural adaptation, and psychometric evaluation of the Patient Assessment Chronic Illness Care tool in Ethiopia (PACIC-5As-ET) for patients with type 2 diabetes
Source: PLoS One. 2026 Jun 11;21(6):e0329197. doi: 10.1371/journal.pone.0329197 (PMC13258013; doi:10.1371/journal.pone.0329197)
Supplement: S3 Table — (DOCX) [file pone.0329197.s003.docx]

**S3 Table: S**ummary table containing both English and Amharic version of the PACIC-5As questionnaire

**Instruction**/ **መመሪያ** ፡ The following questions ask about your experience or perception of managing your diabetes over the past six months (ከዚህ በታች ያሉት ጥያቄዎች ባለፉት ስድስት ወራት ውስጥ ስላገኙት የህክምና እንክብካቤ ልምድ ወይም አስተሳሰብ የሚመለከት መጠይቅ ነው) ።

| When you experience a chronic disease, it can be difficult to remain healthy. We want to learn from you about the healthcare services provided to you by the healthcare team. Such services might include the care your doctors or nurses provided. Your responses will be kept confidential and will not be shared with anyone else (ቆየት ያለ ህመም ሲያጋጥምዎ ጤናማ ሆኖ መቆየት አስቸጋሪ ሊሆን ይችላል. ከእርስዎ የጤና ተንከባካቢ ቡድን ስለ እርስዎ ሁኔታ ስላለው የእርዳታ አይነት መማር እንፈልጋለን። ይህ ምናልባት መደበኛ ህክምና የሚሰጥወትን ሀኪም ወይም ነርስን ሊያካትት ይችላል፡፡ መልሶችዎ በሚስጥር ይጠበቃሉ ለሌላም ለማንም አይነገርም) ። |
| --- |

Over the past six months, when you have been receiving medical services for type 2 diabetes: (ላለፉት ስድስት ወራት ለስኳር ህመም የህክምና አገልግሎት ሲወስዱ፡)

| **No.** | **English questionnaire** | **Amharic questionnaire** |
| --- | --- | --- |
|  | I was asked to share my opinion when we prepared a treatment plan. | የህክምና እቅድ በሚዘጋጅበት ጊዜ ሀሳብዎን እንዲያጋሩ ተጠይቀው ያውቃሉ? |
|  | Have you ever been informed about and encouraged to be aware of and consider different treatment options? | ስለተለያዩ የህክምና አማራጮች እንዲያዉቁና እንዲያስቡቧቸዉ ተደርጎ ያዉቃል? |
|  | Have you ever been informed to report any side effects or outcomes you experience while taking the medication? | መድሃኒቱን በሚወስዱበት ወቅት ሰላጋጠመዎት የጎንዮሽ ጉዳት ወይም ዉጤት ተጠይቀው ያውቃሉ? |
|  | Have you ever provided a written list of things to do to improve your health? | ጤንነትዎን ለማሻሻል ማድረግ የሚገባዎትን ነገር በጽሁፍ ተዘርዝረው ተሰጥተዎት ያውቃል? |
|  | Do you feel satisfied that you have received appropriate follow-up and support? | የተሰጠኝ አገልግሎት የተሟላ ስለሆነ ረክቻለሁ ብለዉ ያስባሉ? |
|  | Have you been guided to understand how the actions you have taken to care for yourself have contributed to your current condition? | እራስዎትን ለመንከባከብ ያደረጓቸዉ ነገሮች አሁን ላሉበት ሁኔታ እንዴት እንዳገዙ እንዲገነዘቡ ተደርጎ ያወቃል? |
|  | Have you been asked to explain the goals of your self-care practices? | ከህመምዎ ጋር በተያያዘ የሚያደርጓቸው እንክብካቤ አላማቸው ምን እደሆነ ተጠይቀው ያውቃሉሁ? |
|  | Have you ever been assisted in setting specific goals to improve your diet and physical activity? | አመጋገብዎትንም ሆነ የአካል ብቃት እንቅስቃሴዎን ለማሻሽል የሚረዳዎትን ዝርዝር አላማዎችን እንዲያወጡ እገዛ ተደርጎልዎት ያዉቃል? |
|  | Have you ever been provided with a copy of your treatment plan? | የህክምና እቅድዎ ቅጅ ተሰጥትዎት ያዉቃል? |
|  | Have you been advised to go to a specific group or specialist who can provide support or assistance to improve or cope with your condition? | ጤናዎት በደንብ እንዲሻሻል ልዩ ድጋፍ ወይም እርዳታ ወደ ሚያደርግልዎት ባለሙያ እንድሄዱ ተደርጓል? |
|  | Have you been asked to explain your thoughts about your health experience in an interview or survey? | ስለጤና ልምድዎ በቃለ ምልልስም ሆነ በዳሰሳ ጥናት ሀሳብዎትን እንዲገልፁ ተደርጎ ያዉቃል? |
|  | Can you confidently say that the doctor or nurse takes your values and traditions into account when providing care? | ዶክተሮቹም ሆነ ነርሶቹ ህክምና አገልግሎት ሲሰጡ ያለዎትን ባህልና ወጎች ግምት ውስጥ ያስገባ ነዉ ብለዉ በእርግጠኝነት መናገር ይችላሉ? |
|  | Do you believe that support has been provided to develop a healthcare plan that you can implement in your daily life? | በእለት ተእለት ህይዎትዎ ማከናወን የሚችሉትን የህክምና እቅድ እንዲያወጡ እገዛ ተደርጎልዎታል? |
|  | Do you think you have been advised to prepare a plan ahead of time so you can take care of yourself, even in challenging situations? | ሙያተኞች በአስቸጋሪ ሁኔታዎችም ቢሆን ራስዎን ለማስታመም እንዲችሉ አስቀድመዉ እቅድ እንዲዘጋጅ ረድተዉኛል ብለዉ ያስባሉ? |
|  | Have you been asked how this chronic disease affects your life? | ያልብዎት የጤና ችግር ህይዎትዎ ላይ ስላደረሰው ተጽኖ ተጠይቀዉ ያዉቃሉ? |
|  | Have you ever been asked about the state of your health after receiving treatment? | ህክምና ከተደረገልዎት በኋላ ጤናዎ በምን ሁኔታ ላይ እንዳለ ለማወቅ ተጠይቀዉ ያዉቃሉ |
|  | Do you think that you have been encouraged to participate in programs within the community that can help you? | በማኅበረሰቡ ውስጥ ሊረዱኝ በሚችሉ ፕሮግራሞች ላይ እንድገኝ ተበረታትቻለሁ ብለው ያስባሉ? |
|  | Have you ever been informed to seek out dietitians, health educators, or counselors? | የስነምግብ ባለሙያ፣ የጤና አስተማሪ ወይም አማካሪ እንዲያገኙ ተልከው ያዉቃሉ? |
|  | Have you been told that being treated by other healthcare professionals like ophthalmologists, surgeons, and others helped you to improve your healthcare or treatment? | በሌሎች የህክምና ባለሙያዎች ለምሳሌ በዓይን ሀኪም፣ በቀዶ ጥገና ሀኪሞች እና በመሳሰሉት መታየት እንዴት ህክምናዎን እንደሚያግዝ ተነግሮዎት ያዉቃል? |
|  | Have you been asked how the care you received from other doctors helped you? | ከሌሎች ሀኪሞች ጋር ያለዎት ክትትል እንዴት እንደሆነ ተጠይቀዉ ያዉቃሉ? |
|  | Have you ever been asked about what topics you would like to discuss with your doctor during your appointments? | በክትትልዎ ስአት ስለምን ማዉራት እንድሚፈልጉ ተጠይቀዉ ያዉቃሉ? |
|  | Have you been asked if you have faced any problems related to self-care in your workplace, with your families, or with social conditions? | ከስራዎ፤ ከቤተሰብዎ ወይም ከማህበራዊ ሁኔታ ጋር በተያያዘ ራስዎን ለመንከባከብ ያጋጠመዎት ችግር መኖሩን ወይም አለመኖሩን ተጠይቀዉ ያዉቃሉ? |
|  | Have you been supported to develop a plan to receive support from your friends, family, and community? | ከጓደኞዎ፣ ከቤተሰብዎ እንዲሁም ከማህበረሰቡ እርዳታ ለማግኘት የሚያስችልዎትን እቅድ ለማዘጋጀት እገዛ ተደርጎልዎት ያዉቃል? |
|  | Have you ever been informed about how important certain self-care activities (e.g., physical exercise) are for your health? | እራስዎትን ለመንከባከብ የሚያደርጓቸዉ ነገሮች (ለምሳሌ የአካል ብቃት እንቅስቃሴ) ለጤናዎ ምን ያህል አስፈላጊ እንደሆኑ ተነግሮዎት ያዉቃል? |
|  | Have you ever set a goal with your healthcare team to manage your health? | ከጤና ሙያተኞች ጋር በመሆን ጤናዎን ለመጠበቅ የሚያስችልዎትን እቅድ አዉጥተዉ ያዉቃሉ? |
|  | Have you been provided a follow-up record or book to monitor your progress or improvements in your health? | ያለዎትን የጤና መሻሻል ለመመዝገብ የሚረዳዎት መዝገብ (ደብተር) ተስጥቶዎት ያውቃል? |
